# Chronic application of alcohol-soluble gluten extract over undamaged skin causes clinical sensitization for life-threatening anaphylaxis via activation of systemic Th2 immune responses in mice

**DOI:** 10.3389/falgy.2023.1214051

**Published:** 2023-09-29

**Authors:** Rick Jorgensen, Haoran Gao, Shivam Chandra, Vaisheswini Sundar, Jaden Loy, Chris Van Antwerp, Perry K. W. Ng, Venu Gangur

**Affiliations:** ^1^ Food Allergy and Immunology Laboratory, Department of Food Science and Human Nutrition, Michigan State University, East Lansing, MI, United States; ^2^ Cereal Science Laboratory, Department of Food Science and Human Nutrition, Michigan State University, East Lansing, MI, United States

**Keywords:** gluten allergy, durum wheat, skin sensitization, systemic anaphylaxis, IgE, Th2, mouse model

## Abstract

**Introduction:**

Gluten allergy is a major public health problem that is growing at an alarming rate. Specific mechanisms underlying sensitization to gluten remain incompletely understood. Currently, it is unclear whether chronic exposure to alcohol-soluble gluten extract via undamaged skin has the capacity to clinically sensitize mice for life-threatening anaphylaxis. Using an adjuvant-free mouse model, here we tested the hypothesis that chronic application of alcohol-soluble durum gluten (ASDG) extract will clinically sensitize mice for life-threatening anaphylaxis.

**Methods:**

This study was conducted in a gluten-free Balb/c mouse colony that was established and maintained on a plant protein-free diet. Groups of adult female mice were exposed dermally to ASDG extract or vehicle once a week for 9-weeks. Specific (*s*) and total (*t*) IgE levels were quantified. Mice were challenged systemically with ASDG to measure symptoms of systemic anaphylaxis. Hypothermic shock response (HSR) and mucosal mast cell degranulation response (MMCR) were determined upon challenge. Spleen Th1, Th2, and other immune markers were quantified.

**Results:**

We found that chronic exposure to ASDG elicited robust elevation of sIgE and tIgE. Systemic challenge with ASDG, but not vehicle, elicited life-threatening anaphylaxis associated with dramatic HSR and MMCR. Correlation analysis demonstrated direct positive inter-relationships among IgE, HSR, and MMCR. Anaphylaxis was associated with significant elevation of prototypic Th2 but not Th1 immune markers in the spleen.

**Discussion/Conclusion:**

Our study collectively demonstrates that ASDG is intrinsically allergenic; and chronic exposure to ASDG via undamaged skin can clinically sensitize mice for life-threatening anaphylaxis via activating the systemic Th2 immune responses.

## 1. Introduction

Gluten is a major group of proteins found in cereal grains such as wheat, barley, and rye. Traditionally, glutens are classified based on their solubility properties into two general groups such as ethanol-soluble prolamin proteins (gliadins, 30%–40% of the total proteins), and weak acid-soluble proteins (glutenins, 45%–50% of the total protein). Non-gluten proteins (being albumins/globulins) are the remainder proteins that are soluble in aqueous solutions (1, 2). Gliadins exist as individual proteins that interact through hydrogen bonds and primarily have intramolecular disulfide bonds. In contrast, glutenins are polymeric proteins that form connections through both intermolecular and intramolecular disulfide bonds. Additionally, gliadins can also be linked to the glutenin network through intermolecular disulfide bonds (3). Within the group of alcohol-soluble proteins, ω−1, 2, 5 gliadin, and α/β/γ-gliadins have been extensively characterized and are known to elicit allergic reactions in susceptible individuals (4, 5).

There is extensive evidence that wheat gluten has the capacity to elicit several immune-mediated diseases. These include gluten hypersensitivity, celiac disease (CD), and non-celiac gluten sensitivity (NCGS) (1). Among these, gluten hypersensitivity (also known as gluten food allergy or wheat food allergy) is potentially deadly (4, 6, 7). Hypersensitivity reactions to wheat are caused by inappropriate activation of immune system by wheat proteins that includes both gluten as well as non-gluten proteins (4, 8).

The underlying immune mechanisms as well as clinical presentations of gluten hypersensitivity are completely different from that of celiac disease (CD) and non-celiac gluten sensitivities (NCGS). Gluten hypersensitivity is primarily due to the production of IgE antibodies against gluten during initial exposures to gluten that sensitizes mast cells. Subsequent exposures to gluten results in mast cell degranulation leading to potentially deadly anaphylactic reactions (3, 9). In contrast, gluten-triggered CD is an autoimmune chronic inflammatory disease that affects primarily the small intestine in most cases; in some cases, gluten also causes celiac disease associated with dermatitis and brain damage (10, 11). The NCGS show up clinically as a chronic digestive disorder caused by unknown mechanisms, although innate immune system activation is implicated (12).

The prevalence of all three gluten-induced disorders have been increasing at an alarming rate worldwide (13, 14). Current estimates of gluten hypersensitivity in the United States are 0.4%–3% (15–17). The prevalence of CD and NCGS are 1% and 3%–6%, respectively (18, 19). Currently there is no cure for these diseases other than a complete avoidance of gluten-containing foods (20). Consequently, gluten-free and wheat-free diets are the primary mode of treatment and management of these diseases.

While clinical sensitization to gluten is required for manifestation of anaphylaxis, the specific mechanisms (for example, route of sensitization) underlying gluten hypersensitivity are incompletely understood at present (21). It is generally presumed that oral exposure to the dietary gluten leads to sensitization resulting in gluten hypersensitivity (1). However, there is also evidence that exposure to gluten can occur via non-oral routes including: skin, airways, and eyes (2, 22–24). Therefore, it is critical to determine whether non-oral exposure to gluten can result in clinical sensitization. It is interesting to note that exposure through skin to food proteins such as tree nuts, sesame, milk, shellfish, etc., has been implicated in sensitization to the respective food hypersensitivities (25, 26). However, whether chronic exposure to gluten via undamaged skin can lead to clinical sensitization is unknown at present.

There are several animal models of gluten hypersensitivity reported in the literature including dogs, rats, guinea pigs and mice (27–32). A common feature of most of these models has been the use of adjuvants to elicit sensitization to gluten. Although such an approach is very popular as it elicits robust sensitizations to gluten, it is not useful when evaluating the intrinsic allergenicity of gluten since adjuvants artificially exacerbate immune responses to gluten. There is one previous mouse model study involving skin exposure to gluten; in this study, mouse skin was deliberately damaged by removing the outer layer of stratum corneum using a tape-stripping method; gluten was applied over the damaged skin along with a detergent to induce sensitization (33). Thus, there are two specific gaps in knowledge in gluten hypersensitivity: (i) whether gluten is intrinsically capable of sensitizing mice without using adjuvants; and (ii) whether chronic application of gluten over undamaged skin can elicit clinical sensitization in mice. These two questions were the foci of this study.

Using an adjuvant-free mouse model, here we tested the hypothesis that chronic application of alcohol-soluble durum gluten (ASDG) extract will clinically sensitize mice for anaphylaxis. There were eight objectives for this study: (1) to establish a colony of gluten-free Balb/c mice; (2) to test the intrinsic sensitization capacity of ASDG when repeatedly applied over undamaged skin by measuring IgE antibody responses; (3) to study clinical symptoms of anaphylaxis upon systemic ASDG challenge; (5) to quantify hypothermic shock responses (HSR); (6) to quantify mucosal mast cell degranulation responses (MMCR); (7) to determine inter-relationships among IgE, HSR, and MMCR; and (8) to characterize the systemic T-helper (Th)-1, and Th2 immune responses in this model.

This study collectively demonstrates that ASDG is intrinsically allergenic, and chronic exposure to ASDG via undamaged skin can clinically sensitize mice for life-threatening anaphylaxis *via* activating the systemic Th2 immune responses.

## 2. Materials and methods

### 2.1. Chemicals and reagents

Biotin-conjugated rat anti-mouse IgE-paired antibodies were purchased from BD BioSciences (San Jose, CA, USA). p-nitro-phenyl phosphate was obtained from Sigma (St Louis, MO, USA). Streptavidin alkaline phosphatase was obtained from Jackson ImmunoResearch (West Grove, PA, USA). Folin reagent was purchased from BioRad (Hercules, CA). The following reagents were obtained as listed: IgE Mouse Uncoated ELISA Kit with Plates; Streptavidin-HRP, TMB substrate; MCPT-1 (mMCP-1) Mouse Uncoated ELISA Kit with Plates; Avidin-HRP, TMB substrate (all from Invitrogen, MA, USA); Tissue Protein Extraction Reagent (T-PER™, a proprietary detergent in 25 mM bicine, 150 mM sodium chloride (pH 7.6) (from ThermoFisher Scientific, MA, USA); protease (serine, cysteine, and acid proteases, and aminopeptidases) inhibitor cocktail (Sigma-Aldrich, MO, USA).

### 2.2. Mice breeding and establishment of a plant-protein-free mouse colony

Adult Balb/c breeder pairs were purchased from The Jackson Laboratory (Bar Harbor, ME).

Once received, the mice were placed on a strict plant-protein-free diet (AIN-93G, Envigo, Madison, MI, USA). Once acclimated after one week, breeding was set up using standard procedure. Adult female mice from the litter (6–8 weeks) were used in this study. All mice were maintained on the strict plant-protein-free diet (AIN-93G) throughout the entirety of the study. All animal procedures were in accordance with Michigan State University policies.

### 2.3. Preparation of alcohol-soluble protein extract from Durum wheat flour

Durum wheat flour (AABB genotype, Carpio variety) was used in protein extraction. Alcohol-soluble durum gliadin was extracted using an Osborne method of sequential extraction (34). Briefly, flour and sterile 0.5 M NaCl in a 1:10 ratio (m/v) were mixed continuously for 2 h followed by centrifugation at 20,000 g for 30 min. Pellets were stored and used in alcohol extraction. Salt-insoluble pellets were then mixed in a 1:10 ratio with 70% ethanol for 2 h followed by centrifugation at 20,000 g for 15 min. The supernatant was frozen at −70°C overnight and freeze-dried the next day. Lyophilized alcohol-soluble durum gluten (ASDG) was reconstituted with 70% ethanol to a concentration of 1 mg protein per 100 uL for topical application. For IP injection challenges, ASDG was reconstituted with phosphate buffered saline (PBS) to a concentration of 0.5 mg/mouse. Protein content was determined using the LECO total combustion method (LECO, St. Joseph, MI, USA). Protein quality was tested by SDS-PAGE.

### 2.4. Skin sensitization, bleeding, and plasma sample preparation

Adult female Balb/c mice were used in experimentation. For transdermal allergen applications, ASDG dissolved in 70% ethanol was used in skin application (100 uL/mouse applied over a 2 cm^2^ area on the rump). For transdermal control applications without allergen, 70% ethanol was used in skin application as a vehicle application (100 uL/mouse over a 2 cm^2^ area on the rump). The application site of allergen or vehicle was over the rump skin of mice, once a week for nine weeks. The rumps of the mice were clipped bilaterally to remove hair (Philips, Amsterdam, Netherlands). The ASDG was applied on the rump at a dose of 1 mg/mouse or vehicle (70% ethanol). The applied area was then covered with a non-latex bandage (Johnson & Johnson, New Brunswick, New Jersey) for one day. This was repeated once a week for a total of nine weeks. Blood was collected before the first exposure and after the 6th exposure via the saphenous vein in anti-coagulant (lithium heparin) coated tubes (Sarstedt Inc MicrovetteCB 300 LH, Germany). The blood was then centrifuged to obtain plasma. Plasma was stored individually at −70°C until needed for testing.

### 2.5. Elicitation of systemic anaphylaxis and clinical symptom scoring

Two weeks after the final skin exposure to ASDG or vehicle mice were challenged via intraperitoneal (IP) injection with either 0.5 mg ASDG or vehicle (PBS). Mice were observed for signs of systemic anaphylaxis during the 30 min after the IP injection as described previously (26). Scores were recorded based on the following criteria: 0 = no symptoms; 1 = scratching and rubbing of the nose and head; 2 = puffiness around the eyes and mouth, diarrhea, pilar erecti, reduced activity and/or decreased activity with a noted increase of respiratory rate; 3 = wheezing, labored breathing, cyanosis around the tail and the mouth; 4 = no activity after prodding, tremors, and convulsions; 5 = death.

### 2.6. Elicitation of oral anaphylaxis

Two weeks after the final exposure to ASDG, mice were orally challenged with ASDG (20 mg per mouse in 300 µL sterile saline) using curved feeding needles (22-gauge, length: 1.4 in, Kent Scientific, Torrington, CT, United States) as reported previously (35).

### 2.7. Determination of hypothermic shock responses

Rectal temperature (°C) was recorded before the challenge and every 5 min after the challenge, up to 30 min using a rectal thermometer (DIGI-SENSE, MA, USA). Actual temperatures and the change in rectal temperature (Δ°C) at every 5-minute increment compared to the before-challenge temperatures for each respective mouse were utilized in analyses.

### 2.8. Measurement of specific IgE antibody levels

The ASDG-specific (s) IgE antibody levels were measured using a previously reported ultrasensitive ELISA method with modifications (21, 36, 37). Briefly, 96-well Corning 3,369 plates were coated with ASDG followed by blocking with 5% gelatin. The plate was then washed, plasma sample was added, washed again, biotin-conjugated anti-mouse IgE antibody was added, washed, addition of streptavidin alkaline phosphatase and finally p-nitro-phenyl phosphate to allow for quantification as previously reported (36, 37). Mice samples were screened individually in quadruplicate.

### 2.9. Measurement of specific IgG1 antibody levels

The levels of ASDG-specific IgG1 antibodies were measured using a modified ultrasensitive ELISA method previously reported (37, 38). In brief, 96-well Corning 3,369 plates were coated with ASDG and blocked with bovine serum albumin. Subsequently, the plate was washed, and plasma samples were added. After another wash, biotin-conjugated anti-mouse IgG antibodies were added, followed by additional washing steps. Streptavidin alkaline phosphatase was then added, and p-nitro-phenyl phosphate was used for quantification, as previously described (37, 38).

### 2.10. Measurement of total plasma IgE concentration

Total (*t*)IgE concentration was determined using a commercial ELISA kit (Invitrogen, Waltham, MA, USA). Briefly, 96-well Corning Costar 9,018 plates were coated with capture antibody (anti-mouse IgE) followed by the addition of plasma samples and standards (recombinant mouse IgE). A secondary antibody (anti-mouse IgE) and then a detection system of Streptavidin-HRP and TMB substrate was added as described previously (36, 37). Assay limit of detection is 4 ng/ml. The standard range used for analysis is 4–250 ng/ml. Samples were tested individually from each mouse in quadruplicate.

### 2.11. Quantification of mucosal mast cell protease-1 (MMCP-1) level

Blood was collected one hour after challenge and was used in measurement of mucosal mast cell protease-1 levels (MMCP-1) in the plasma using an ELISA-based method developed by Invitrogen as described previously (36, 37). Briefly, 96-well Corning Costar 9,018 plates were coated with capture antibody (anti-mouse MMCP-1) followed by the addition of samples and standards (recombinant mouse MMCP-1). Biotin-conjugated anti-mouse MMCP-1 was then added as a secondary antibody. Detection was completed utilizing an avidin-HRP/TMB substrate system. The limit of detection for this assay is 120 pg/ml and the standards ranged from 120 to 15,000 pg/ml. Individual mouse plasma was tested in quadruplicate.

### 2.12. Spleen extract preparation and analysis of immune markers

After one-hour post-challenge, mice were euthanized, and the spleens were harvested. The spleens were snap-frozen in liquid nitrogen and stored at −70°C. Tissue was extracted as described previously (21). Briefly, spleen tissue was immersed in a tissue protein extraction reagent (T-PER) buffer with protease inhibitor. For every 100 mg of tissue, 10 µL of protease inhibitor per 1 ml T-PER buffer was used. Ultra-sonication was utilized to homogenize the spleen tissue for two 30-second intervals with a resting period of 5 min in between. After the second homogenization, the samples were rested for 15 min and placed for centrifugation (13,500 × g) at 4°C for 10 min. The supernatant was then collected and aliquoted for storage at −70°C. Immune markers were quantified by RayBiotech service utilizing the Quantibody microarray (RayBiotech, Atlanta, GA, USA) (https://www.raybiotech.com/mouse-cytokine-array-q2000/). Samples were analyzed in quadruplicate.

### 2.13. Histopathology

The formalin-fixed skin tissues underwent processing at the Michigan State University Histopathology and Cytology Service Laboratory using well-established methods as described previously (36, 39). Paraffin sections were prepared using the Reichert Jung 2030 rotary microtome and stained with Hematoxylin and Eosin.

### 2.14. Quantification of plasma histamine levels

Histamine concentration was determined using a commercial ELISA kit (IBL America, MN, USA). Briefly, acylated standards, controls, and samples, along with the solid phase-bound analyte, compete for limited antiserum binding sites. After reaching equilibrium, free antigen and antigen-antiserum complexes are washed away. The antibody bound to the solid phase is then detected using an anti-rabbit IgG-peroxidase conjugate, with TMB serving as the substrate for the enzymatic reaction. The resulting color change was measured at 450 nm. The concentration of unknown samples was determined by comparing their absorbance with a reference curve generated using known standard concentrations. Assay limit of detection was 0.2 ng/ml. The standard range used for analysis was 0–50 ng/ml.

### 2.15. Statistics

An online software service was used in these analyses (https://www.socscistatistics.com/tests/). A student's *t*-test was used to compare two groups, and ANOVA was used for multiple group comparisons. The statistical significance level was set at *p* < 0.05. Pearson correlation coefficient calculation was conducted using excel built-in program. The following formula was used to calculate *r*-scores:

$$r=\frac{\sum \left(x-\bar{x})(y-\bar{y}\right)}{\sqrt{\sum {\left(x-\bar{x}\right)}^{2}\sum {\left(y-\bar{y}\right)}^{2}}}$$

Using the *r*-scores, and *n*-values significance was calculated with *p* < 0.05.

## 3. Results

### 3.1. Chronic application of alcohol-soluble durum gluten (ASDG) onto undamaged skin elicits robust specific IgE antibody response in Balb/c mice

**Figure 1:**
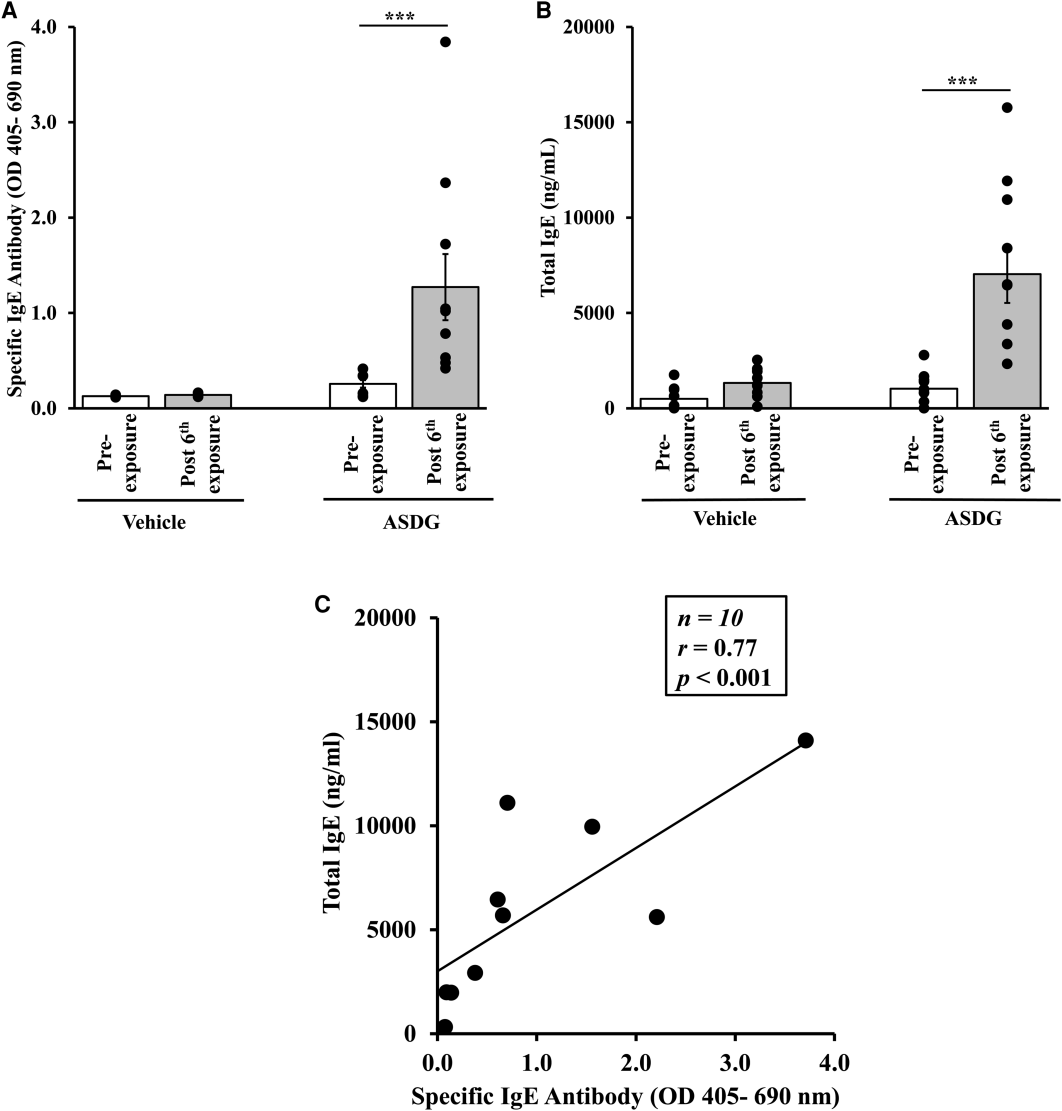
Chronic skin exposure to alcohol-soluble durum gluten (ASDG) elicited specific IgE antibody responses and elevation of total IgE in balb/c mice. Mice were exposed to ASDG (1 mg in 100 µL 70% ethanol/mouse, *n* = 10) or to vehicle (70% ethanol, 100 µL/mouse, *n* = 10) as described in Materials and Methods. Blood was collected before 1st exposure (Pre) and after 6th exposure. Plasma was used in measurement of ASDG-specific IgE levels (OD 405–690 nm) using an ELISA method described previously. (**A**) ASDG-specific IgE antibody levels in vehicle-exposed mice and ASDG-exposed mice before and after 6th exposure. (**B**) Total IgE levels in vehicle-exposed mice and ASDG-exposed mice before and after 6th exposure. Student's two-tailed *t*-test: ***p < 0.0001. (**C**) Pearson correlation analysis between alcohol-soluble durum gluten (ASDG)-specific IgE antibody levels and total IgE levels. Pearson correlation analysis was used to test the relationship between ASDG-specific IgE antibody and total IgE levels in the plasma after 6th transdermal exposure to ASDG.

The ability of ASDG to elicit sensitization upon chronic application over the skin was tested as follows: Groups of female adult mice (*n *= 10/group) were exposed topically to ASDG or to vehicle once per week for nine weeks as described in the methods. Specific (*s*) IgE levels were measured in blood collected before (pre) exposure and after 6 skin exposures using an ELISA method pre-optimized for this purpose. As shown in Figure 1A, skin exposure to ASDG, but not vehicle elicited robust sIgE response. Serial two-fold dilution of plasma sample demonstrated specific IgE levels at 1/320 dilution (Supplementary Figures 1).

### 3.2. Chronic application of alcohol-soluble durum gluten (ASDG) onto undamaged skin also elevates total IgE levels, which correlate with sIgE levels

Total IgE levels in the blood collected before (pre) exposure and after exposure to ASDG or vehicle were measured by ELISA method. As evident, chronic exposure to ASDG significantly elevated tIgE levels, which were not noted in vehicle-exposed mice (-7-fold increase in ASDG-exposed mice vs. vehicle-exposed mice) (Figure 1B). Pearson correlation coefficient analysis was conducted using individual mouse data for sIgE and tIgE. A significant positive correlation was found between the two readouts (Figure 1C).

### 3.3. ASDG-sensitized, but not vehicle-sensitized mice, exhibit life-threatening symptoms of anaphylaxis upon systemic challenge with ASDG

**Figure 2:**
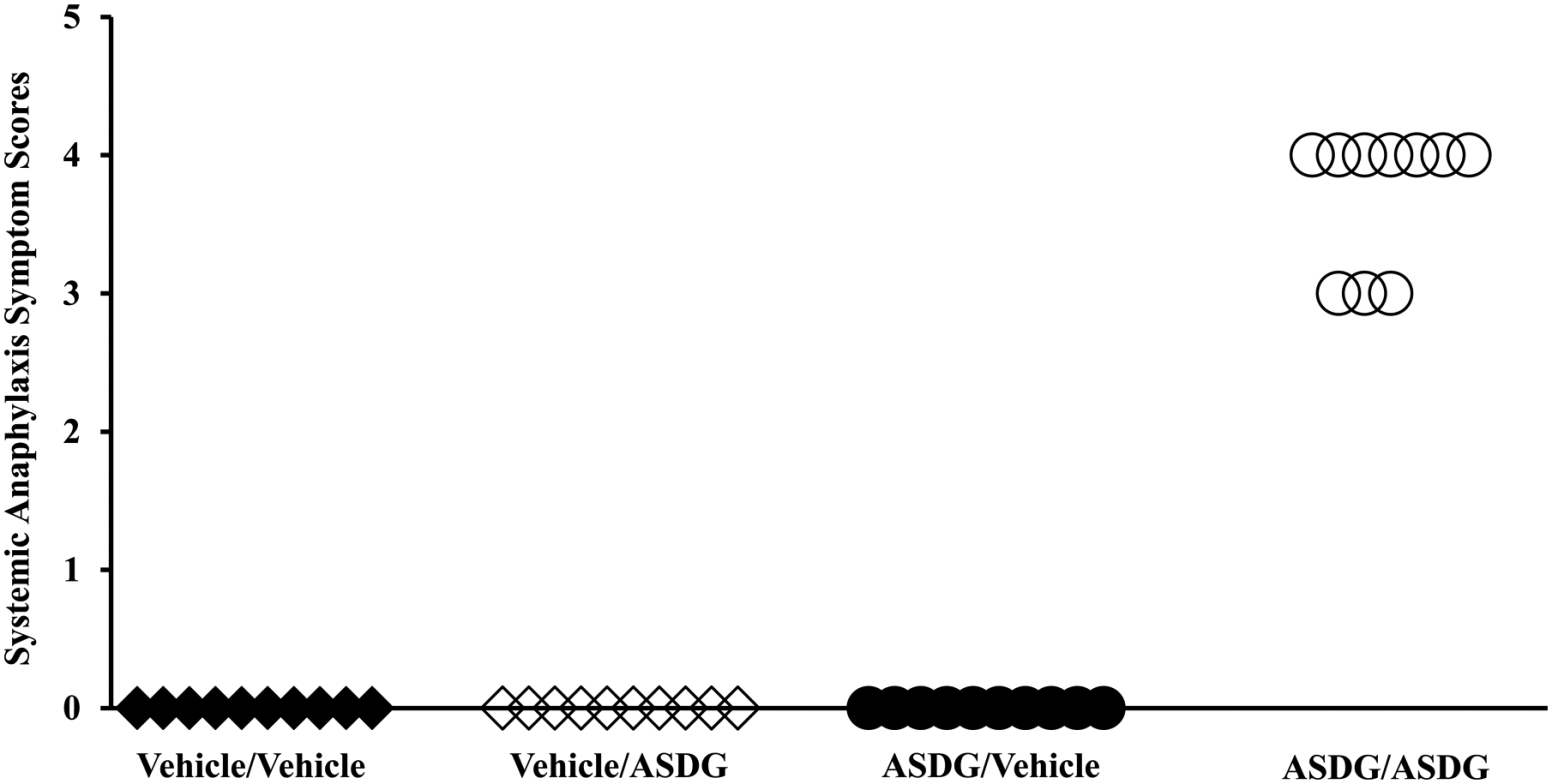
Chronic skin exposure to alcohol-soluble durum gluten (ASDG) is sufficient to clinically sensitize balb/c mice for systemic anaphylaxis. Groups of Balb/c mice were sensitized with either vehicle or ASDG as described in Methods. After 9 transdermal exposures mice were challenged with either vehicle or ASDG (0.5 mg/mouse) by intraperitoneal injection. Mice were monitored for symptoms of systemic anaphylaxis using a published method described in the text. Each symbol represents an individual mouse. Horizontal axis shows different groups as follows: Vehicle/Vehicle = skin exposure with vehicle followed by vehicle challenge; Vehicle/ASDG = skin exposure with vehicle followed by ASDG challenge; ASDG/Vehicle = skin exposure with ASDG followed by vehicle challenge; ASDG/ASDG = skin exposure with ASDG followed by ASDG challenge.

The parallel groups of mice skin-sensitized with ASDG vs. vehicle-only were challenged with intra-peritoneal ASDG to evaluate for systemic anaphylaxis. Clinical symptom scores were determined using published methods (26). As evident, severe life-threatening clinical symptoms were noted only in the ASDG-sensitized mice but not in vehicle groups (Figure 2). Most common symptoms included altered respiration, scratching, and rubbing of nose, face, and/or head, and lack of activity upon prodding.

### 3.4. Mice with systemic anaphylaxis symptoms upon systemic challenge with ASDG exhibit dramatic hypothermic shock responses (HSR)

**Figure 3:**
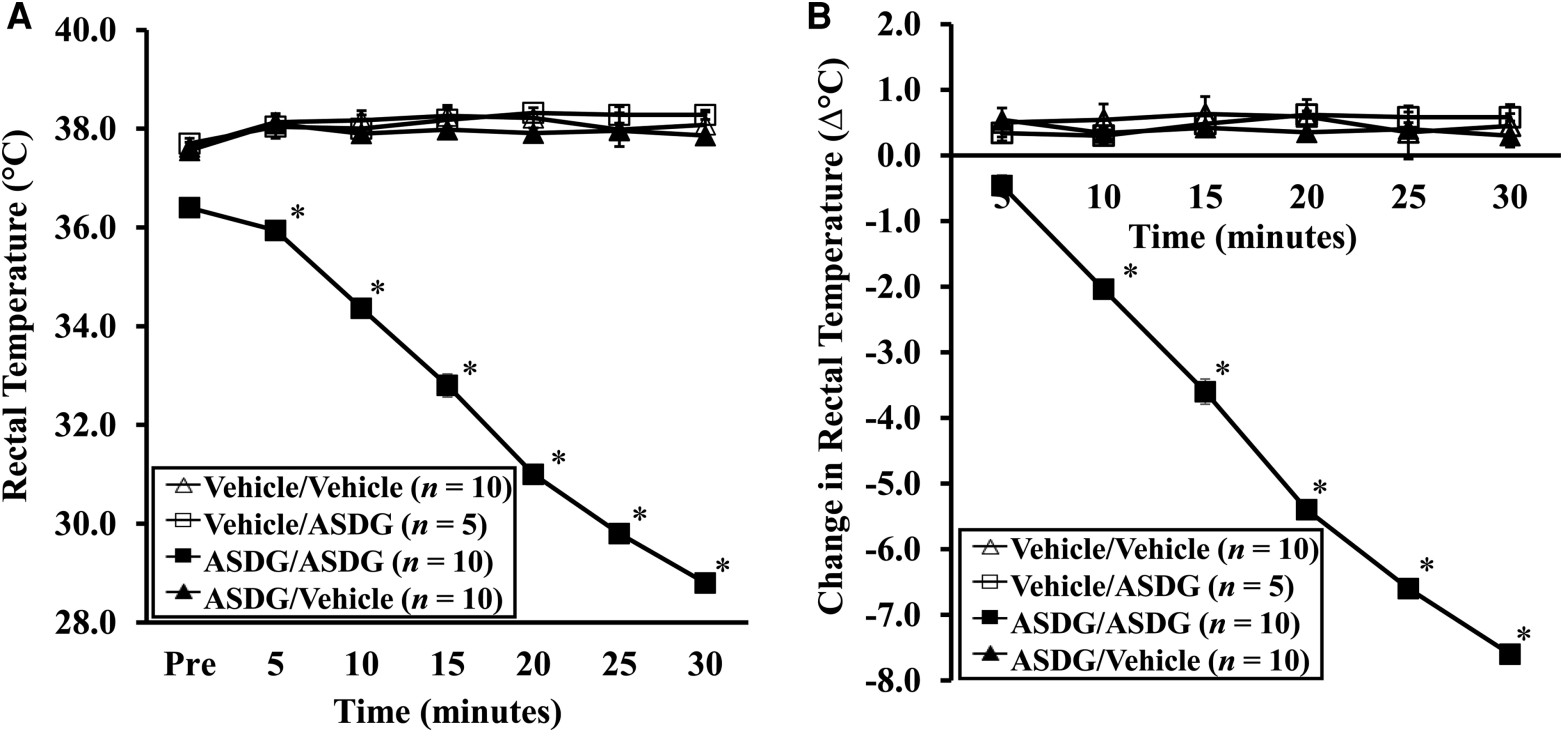
Induction of hypothermic shock responses upon systemic challenge with alcohol-soluble durum gluten (ASDG). Mice exposed to ASDG or to vehicle were systemically challenged by intraperitoneal injection as described in Materials and Methods. (**A**) Rectal temperatures (°C) at indicated time points in vehicle-sensitized and ASDG-sensitized mice challenged with ASDG or vehicle. (**B**) Change in rectal temperature (Δ°C) at indicated time points in vehicle-sensitized and ASDG-sensitized mice challenged with ASDG or vehicle. Vehicle/Vehicle = skin exposure with vehicle followed by vehicle challenge; Vehicle/ASDG = skin exposure with vehicle followed by ASDG challenge; ASDG/Vehicle = skin exposure with ASDG followed by vehicle challenge; ASDG/ASDG = skin exposure with ASDG followed by ASDG challenge. Student's two-tailed *t*-test: *p < 0.05.

Anaphylactic reactions were also quantified using rectal thermometry to evaluate for any hypothermic shock responses (HSR). As evident, systemic challenge with ASDG resulted in life-threatening HSR of ASDG-sensitized, but not of vehicle control mice (Figures 3A–B). Actual temperature changes are shown in Figure 3A. Absolute change in temperature is depicted in Figure 3B. There was no HSR observed in vehicle-challenged mice or in vehicle-sensitized mice challenged with ASDG.

### 3.5. Hypothermic shock responses elicited by ASDG correlates significantly with sIgE levels

**Figure 4:**
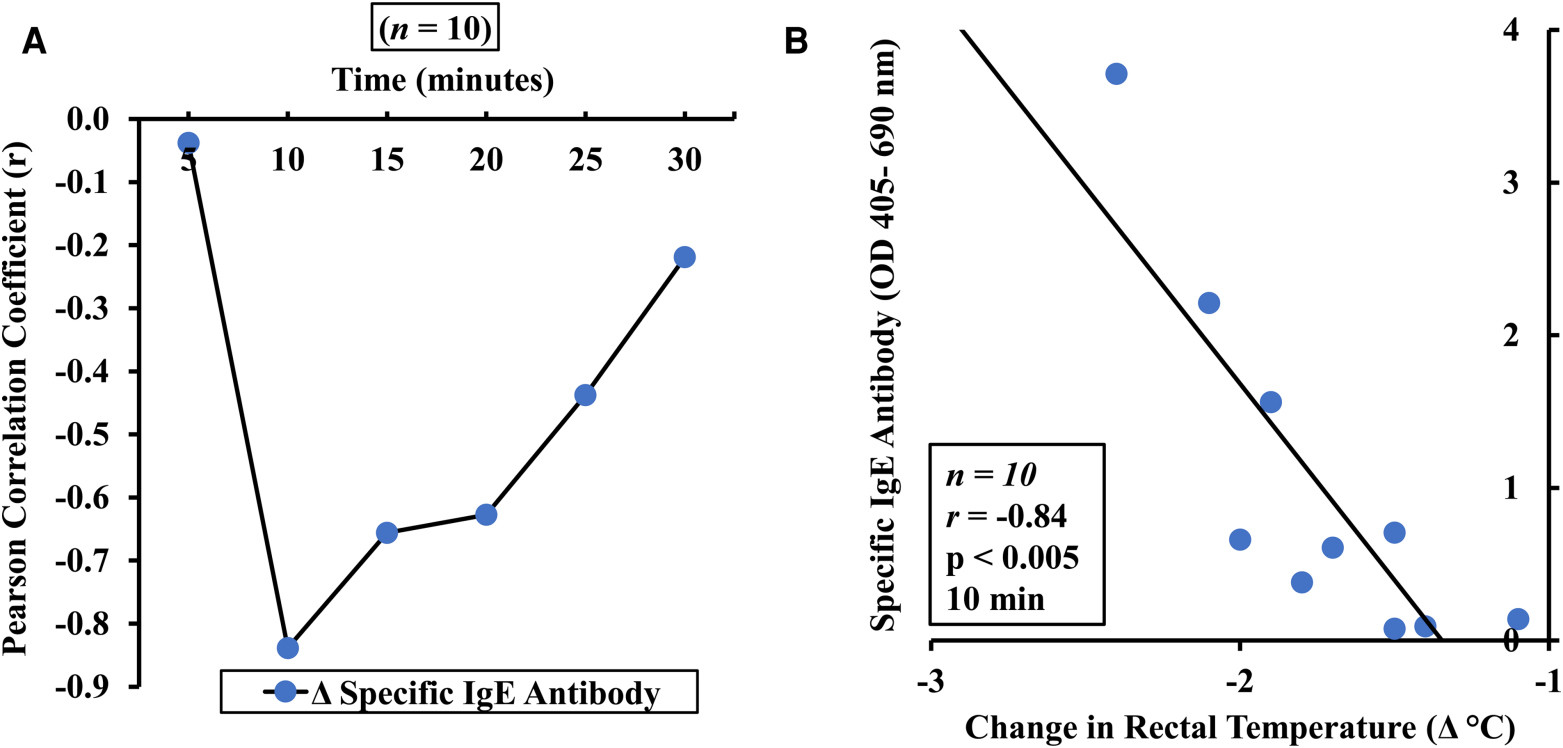
Pearson correlation analysis of hypothermic shock response and alcohol-soluble durum gluten (ASDG)-specific IgE antibody levels in this model. Mice were exposed to ASDG or vehicle and challenged as described in Materials and Methods. Pearson correlation coefficient (*r*) between ASDG-specific antibody levels and change in rectal temperature (Δ°C) in mice sensitized and systemically challenged with ASDG (0.5 mg/mouse). (**A**) Pearson correlation coefficient (*r*) at indicated time points. (**B**) Pearson correlation analysis between ASDG-specific IgE antibody levels and Δ°C at 10 min post challenge.

In order to determine the relationship between the hypothermic shock response and specific IgE response we conducted Pearson correlation analysis. The rationale underlying this was to test whether specific IgE antibody was involved in causing temperature drop at a specific time point. The results are shown in Figure 4. As evident, except for 5 min, temperature drops were significantly correlated with IgE levels at all other timepoints suggesting that IgE mediated allergic reactions likely contributed to the observed temperature drops.

### 3.6. Systemic anaphylaxis is also associated with significant mucosal mast cell degranulation in this model

**Figure 5:**
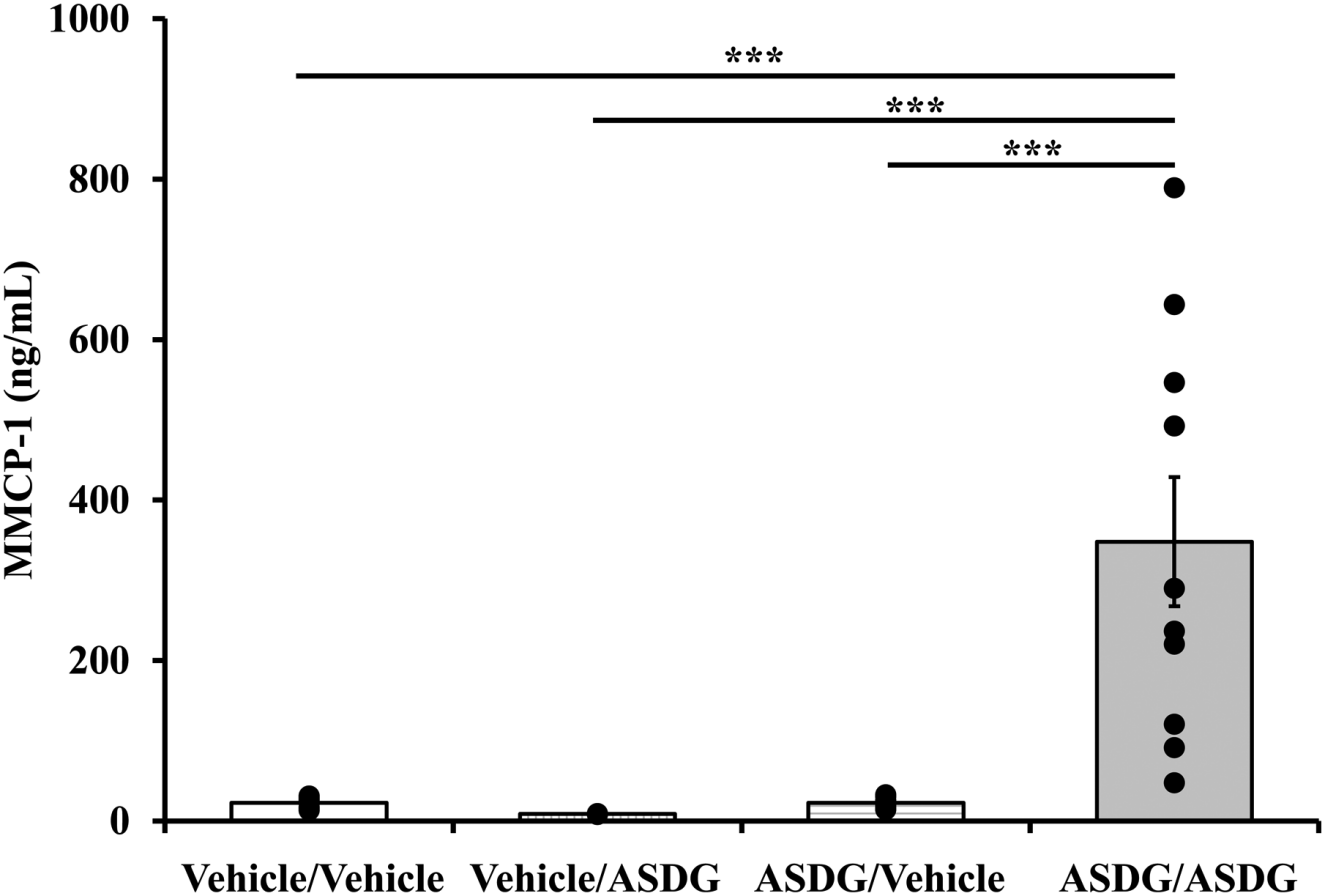
Systemic anaphylaxis induced by alcohol soluble durum gluten (ASDG) is associated with degranulation of mucosal mast cells in this model. Mice were treated as described in Materials and Methods. Their plasma mucosal mast cell protease-1 (MMCP-1) levels (ng/ml) were measured using an ELISA-based method described in the text. Vehicle/Vehicle = skin exposure with vehicle followed by vehicle challenge; Vehicle/ASDG = skin exposure with vehicle followed by ASDG challenge; ASDG/Vehicle = skin exposure with ASDG followed by vehicle challenge; ASDG/ASDG = skin exposure with ASDG followed by ASDG challenge. *** *p* < 0.005, one-way ANOVA and Tukey's *post hoc* tests.

Blood was collected one hour after intraperitoneal challenge and used to measure the mucosal mast cell degranulation response (MMCR) in mice. The elevation of MMCP-1 in the blood demonstrates a true IgE antibody-mediated type-1 hypersensitivity reaction to food proteins in mouse models as described before (40). As seen in Figure 5, significant MMCR is seen in mice undergoing anaphylaxis; no such MMCR was noted in control mice.

### 3.7. Correlation analysis between MMCR and HSR

**Figure 6:**
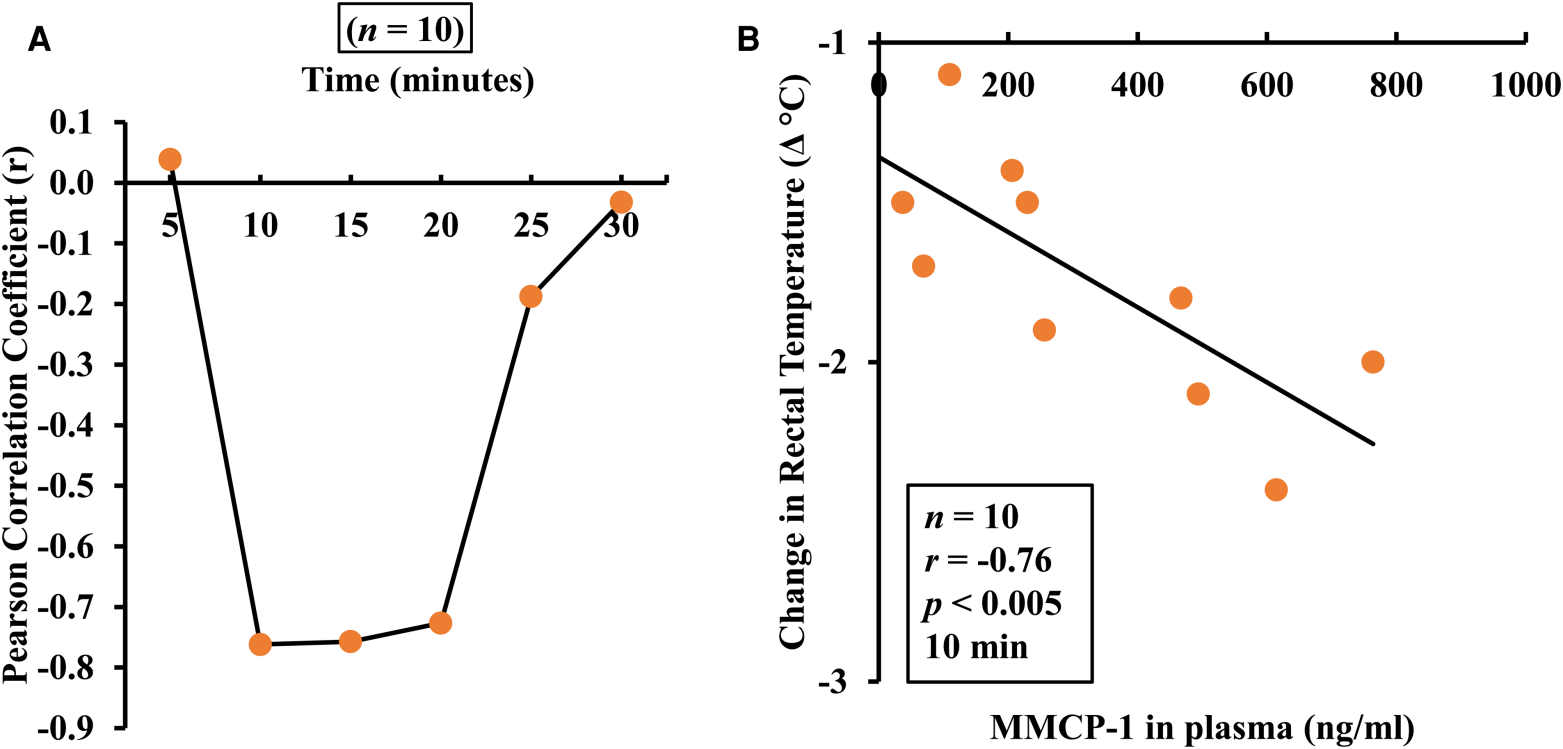
Pearson correlation analysis between hypothermic shock response and mucosal mast cell degranulation in this model. Mice were exposed to alcohol soluble durum gluten (ASDG) or vehicle and challenged as described in Materials and Methods. Pearson correlation coefficient (*r*) between mucosal mast cell degranulation (MMCP-1) and change in rectal temperature (Δ°C) in mice sensitized and systemically challenged with ASDG (0.5 mg/mouse). (**A**) Pearson correlation coefficient (*r*) at indicated time points. (**B**) Pearson correlation analysis between mucosal mast cell degranulation and Δ°C at 10 min post challenge.

Pearson correlation coefficient analysis was conducted using individual mouse data for absolute change in rectal temperatures and for MMCP-1. A significant positive correlation was observed between the two readouts that sustained from 10 to 20 min time points (Figures 6A–B).

### 3.8. Correlation analysis between MMCR and IgE levels

**Figure 7:**
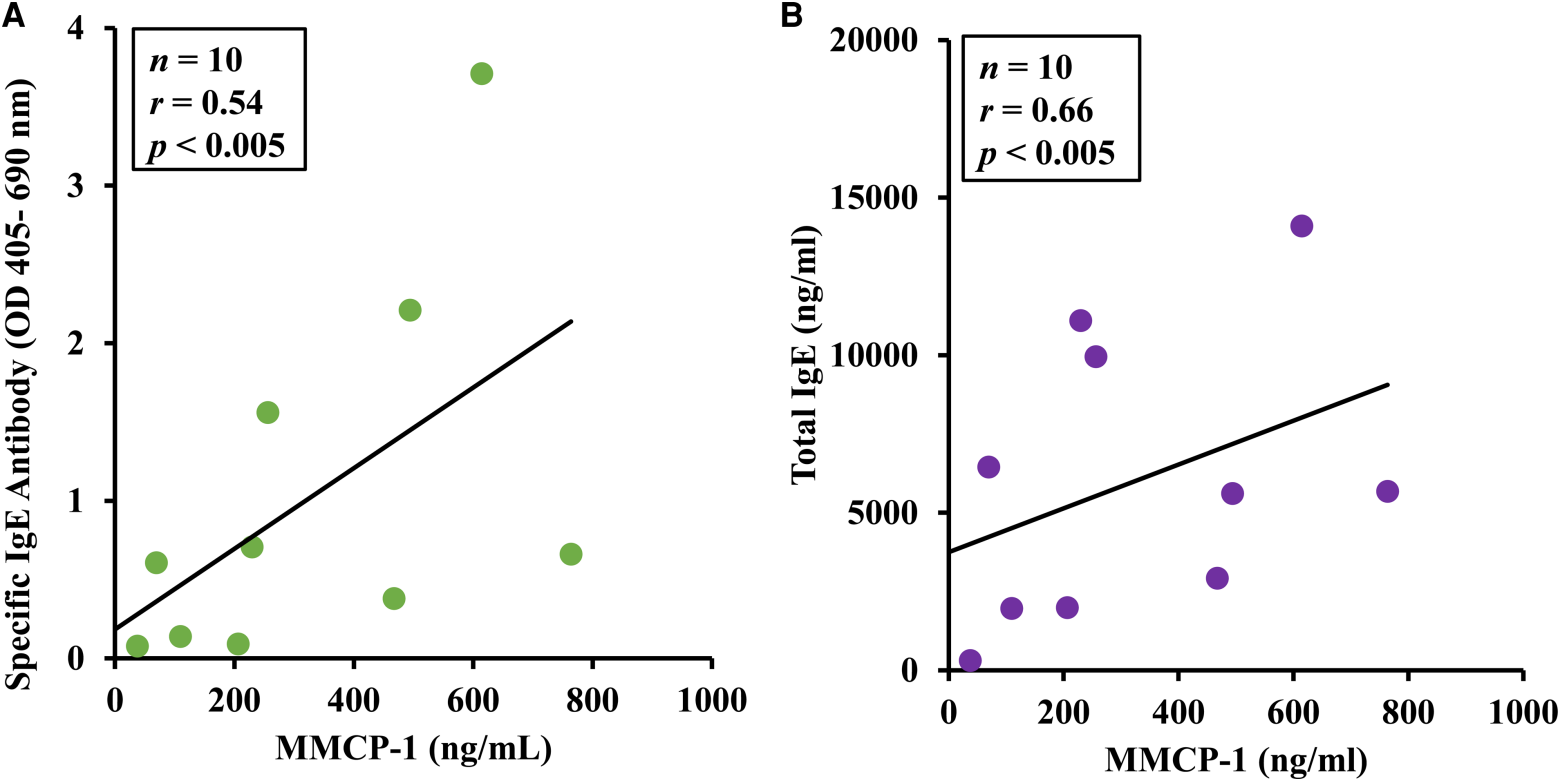
Pearson correlation analysis of IgE responses and mucosal mast cell degranulation responses in this model. Mice were treated as described in Materials and Methods. Pearson correlation analysis was used to test the relationship between mucosal mast cell protease-1 (MMCP-1) levels (ng/ml) and IgE responses (specific IgE antibody or total IgE) in the plasma after 6th transdermal exposure to alcohol soluble durum gluten (ASDG). (**A**) Pearson correlation analysis between ASDG-specific IgE antibody levels and MMCP-1 levels in systemically challenged mice. (**B**) Pearson correlation analysis between total IgE antibody levels and MMCP-1 levels in systemically challenged mice.

Pearson correlation coefficient analysis was conducted using individual mouse data for IgE and MMCP-1. A significant positive correlation was noted between the two readouts (Figures 7A–B).

### 3.9. Characterization of systemic Th1/Th2, and Th17 cytokine responses in the spleen

**Figure 8:**
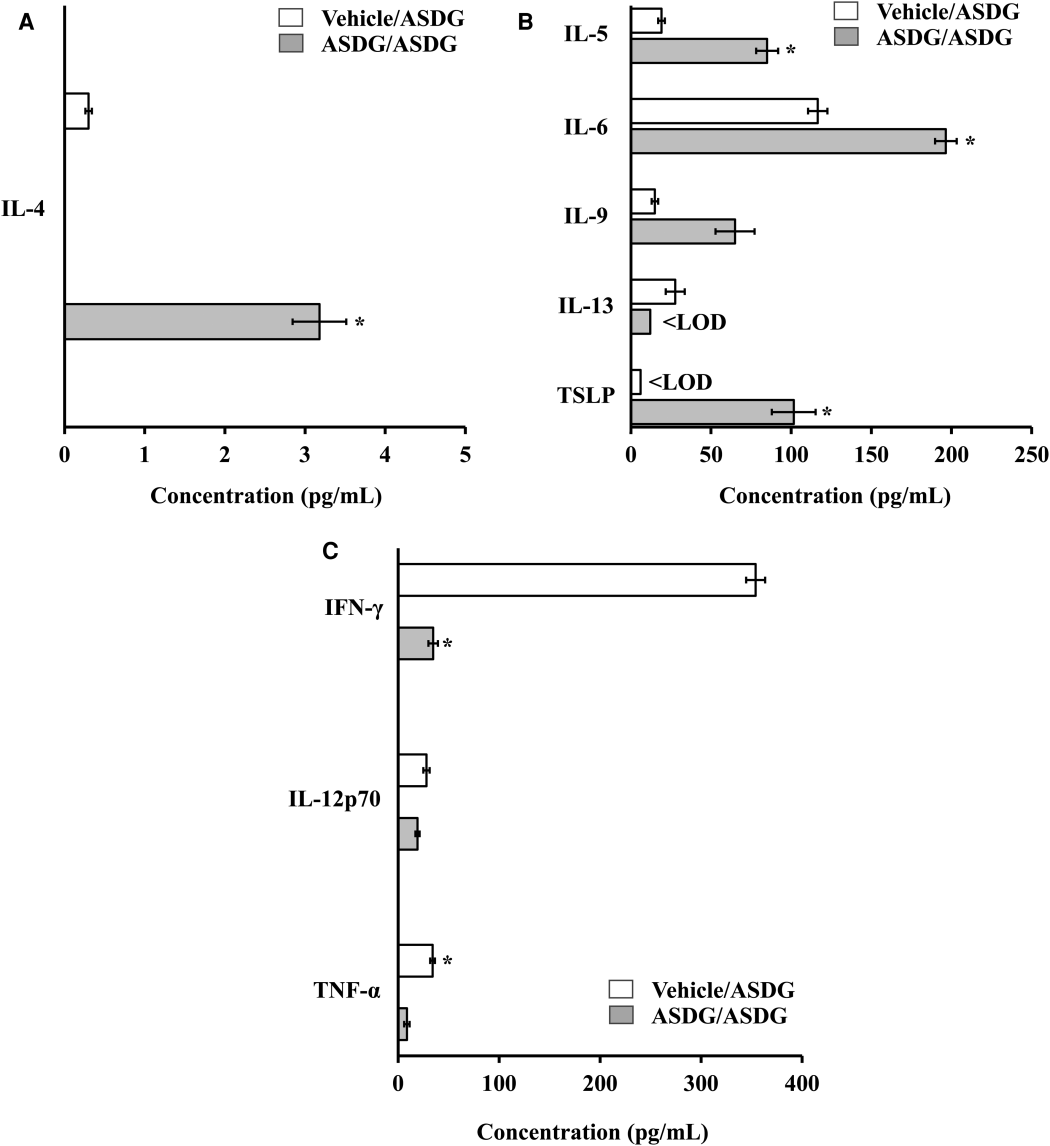
Effect of systemic challenge with alcohol-soluble durum gluten (ASDG) on spleen cytokine responses *in vivo* in vehicle-sensitized control mice vs. ASDG-sensitized allergic mice. Spleen tissues were collected from the experimental groups at 1-h post challenge with ASDG and used in cytokine protein analysis (**A–E**) as described in the methods section. Absolute concentrations of various cytokines are shown (pg/mL).

Spleen biomarkers of Th1, and Th2 immune responses were analyzed. Fold change was determined using mean values. As evident, Th2 cytokines IL-4, IL-5, IL-6, and TSLP were substantially elevated in mice undergoing anaphylaxis (Figures 8A–B). Among Th1 markers, IFN-*γ* and TNF-α were significantly down-regulated; other markers were not substantially altered (Figure 8C). Among Th17 cytokines, IL-17A and IL-23 were significantly increased (Figures 8D–E). A panel of chemokines, and adhesion molecules were also analyzed, and the results are shown in Table 1. As evident, a number of these markers were substantially elevated (ranging from 2.5-fold to 43-fold) in the mice that had systemic anaphylaxis (Table 1).

**Figure:**
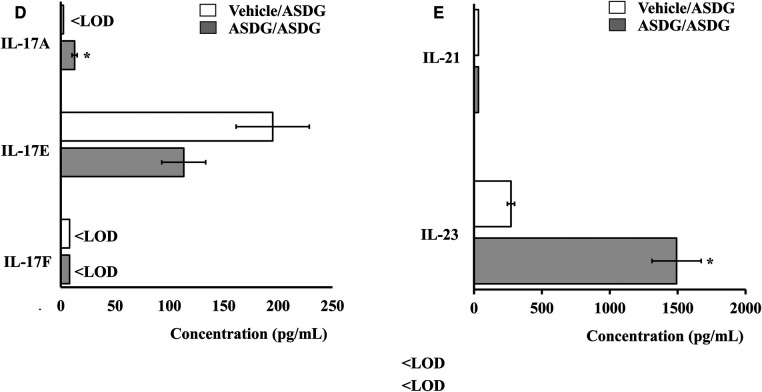


**Table 1:** Chemokines and adhesion molecule in the spleen associated with systemic anaphylaxis induced by alcohol-soluble durum gluten (ASDG) in skin-sensitized mice.

Biomarker^a^	Vehicle-sensitized/ASDG-challenged mice (*n* = 5)	ASDG-sensitized/ASDG-challenged mice (*n* = 5)	Fold-change
Chemokines			
I-TAC (CXCL11)	<2^b^	86.19 ± 38.3	43.094
KC (CXCL1)	32.79 ± 3.03	92.74 ± 3.89	2.828
MCP-1 (CCL2)	18.8 ± 4.9	214.24 ± 25.05	11.393
MIG (CXCL9)	508.27 ± 11.2	1,409.06 ± 114.65	2.772
MIP-3a (CCL20)	<2^a^	22.35 ± 4.65	11.176
MIP-3b (CCL19)	27.03 ± 1.18	97.31 ± 12.63	3.599
Adhesion molecule			
MadCAM-1	778.18 ± 46.16	1,976.45 ± 164.17	2.540

^a^ Spleen immune marker protein contents are shown as pg/ml, mean ± SE.

^b^ Below limit of detection.

### 3.10. Induction of systemic anaphylaxis upon oral challenge with ASDG in this model

Oral challenge with ASDG to ASDG-sensitized mice lead to a significant drop in rectal temperature as shown (Supplementary Figures 2).

### 3.11. Chronic application of alcohol-soluble durum gluten (ASDG) onto undamaged skin elicits robust specific IgG1 antibody response in Balb/c mice

Specific IgG1 levels were measured in blood collected before (pre) exposure and after 6 skin exposures using an ELISA method pre-optimized for this purpose. As shown (Supplementary Figures 3), skin exposure to ASDG elicited robust IgG1 antibody response with 1 in 1,024,000 dilution of plasma showing positive reaction in the assay.

### 3.12. Chronic application of vehicle or alcohol-soluble durum gluten (ASDG) caused no major damage to the skin

Skin tissues from the site of application of ASDG and vehicle were stained with H and E. Microscopic examination of skin samples from the site of application of vehicle or ASDG was conducted. As shown, neither application caused any marked skin damage as evidence by intact stratum corneum (Supplementary Figures 4).

### 3.13. Systemic anaphylaxis upon intraperitoneal challenge with ASDG, but not vehicle, is associated with marked elevation of plasma histamine levels

Histamine elevation in the blood upon systemic challenge with ASDG or vehicle was measured using an ELISA method. As shown, dramatic elevation of histamine was noted only in ASDG-sensitized mice challenged with ASDG (Supplementary Figures 5).

## 4. Discussion

Wheat allergies are potentially deadly, and the mechanisms underlying the development of clinical sensitization for life-threatening systemic anaphylaxis are incompletely understood at present. Both gluten and non-gluten wheat allergens have been implicated in wheat allergies. This study evaluated whether skin exposure to wheat gluten acts as a potential route for deadly immune sensitization. Here we tested the hypothesis that chronic exposure to alcohol-soluble durum wheat gluten (ASDG) extract via skin without artificial skin damage and without co-exposure to adjuvant will clinically sensitize mice for life-threatening anaphylaxis. Our data collectively supports this hypothesis.

There are 8 novel findings from this study: (i) chronic skin exposure to ASDG extract results in progressive increase in systemic levels of anti-ASDG-specific IgE antibody; (ii) chronic skin exposure to ASDG extract also elevates total (t)IgE levels in the blood that significantly correlates with sIgE levels; (iii) mice with chronic skin exposure to ASDG, but not to vehicle, exhibit life-threatening clinical symptoms of anaphylaxis upon systemic exposure to ASDG via intraperitoneal injection; (iv) clinical symptoms of anaphylaxis are associated with a dramatic hypothermia shock response (HSR); (v) anaphylaxis is associated with a significant gut mucosal mast cell degranulation response (MMCR); (vi) single mouse data analysis demonstrates significant correlations among sIgE, HSR and MMCR; (vii) clinical symptoms of anaphylaxis are associated with significant elevations of prototypic Th2 cytokines in the spleen; and (viii) several other novel immune biomarkers associated with gluten-induced anaphylaxis are identified in this model.

To qualify as an allergen, a protein should exhibit binding affinity to serum IgE (41). The protein must also possess two distinct types of epitopes: those that interact with IgE antibodies and those that bind to Th2 cells (42). The generation of antigen-specific IgE antibodies involves the activation of antigen presentation by professional antigen-presenting cells (APCs) like dendritic cells, macrophages/monocytes, and B cells via the *T* helper (Th)-2 immune response (2). In instances of skin sensitization, the protein must also have the ability to activate local Langerhans cells upon breaching the skin barrier. Notably, alcohol could potentially facilitate this process by aiding in skin penetration. Similarly, within the gastrointestinal tract, alcohol might play a role in aiding the breach of the epithelial barrier by substances like gluten, enabling their interaction with immune system APCs in the gut, as gluten epitopes have been known to trigger celiac disease upon consumption of commercial beers (43). After the production of the antigen-specific IgE antibodies, and the subsequent fixation of IgE to FcεR1 receptors on mast cells and basophils, re-exposure to wheat allergens can initiate and propagate life-threatening anaphylaxis upon degranulation of mast cells and basophils (2).

Routes of exposure to wheat gluten as possible mechanisms of sensitization of individuals to gluten resulting in hypersensitivity have been explored for many decades (22–24). Thus, chronic oral and nasal exposures to wheat gluten are generally thought to lead to gluten food allergy and allergic airways disease, including bakers' asthma in genetically susceptible subjects (44–47). However, potential clinical consequences of chronic skin exposure to wheat gluten have been understudied and underappreciated. There is a strong rationale to evaluate this possibility. Human exposure to wheat gluten via skin is expected in the baking industry, and in families. Furthermore, gluten is also used as an ingredient in several cosmetic and skin health products. There are also several reports of life-threatening human clinical sensitizations to gluten via cosmetic such as shampoos and facial soaps (48–51). Thus, it is very critical to evaluate and characterize the immune and clinical consequences of repeated skin exposures to gluten as there is significant recent evidence supporting the general hypothesis that skin exposures to environmental agents have health consequences (52–57).

Specific structural and functional properties required to determine protein allergenicity is a topic of intense research (42). Accordingly, it is important to determine the structural and functional properties of ASDG that makes it a powerful allergen. We speculate the following to explain why ASDG is potently allergenic: (i) ASDG possess highly potent multiple IgE binding epitope structures; for instance previous studies demonstrated presence of several IgE epitopes in the N-terminal domains of α- and γ-gliadins, although both N and C-terminal domains contain IgE epitopes, and the disulfide bonds appear to be of high importance for IgE binding to the epitopes (58). Interestingly IgE epitope structure of gluten for human wheat allergenicity appear to be very similar to that in Balb/c mice model (59); (ii) ASDG also possesses very potent T cell epitopes that drive Th2 immune responses: previous study have shown that gluten elicits powerful Th2 cytokine responses in Balb/c model as evidenced by robust recall IL-4 and IL-5 responses of spleen cells from gliadin sensitized mice upon short-term culture with gliadin allergen *ex vivo* (33); (iii) in the context of skin exposure method we have developed here, we speculate that alcohol-solubility of gluten is a critical factor required for enabling skin penetration by gliadin and making it accessible to the Langerhan's dendritic cells of the skin to activate systemic Th2 response; our date support this hypothesis. Similarly, within the gastrointestinal tract also, alcohol-solubility of gluten might promote easy access of ASDG to the gut dendritic cells triggering Th2 responses.

We used alcohol-solubilized gluten in this study for the development of an experimental model of skin sensitization followed by anaphylaxis disease elicitation. We hypothesize that alcohol solubility of gluten is an important contributor to make immunogenic and allergenic gluten epitopes available to the immune system to generate allergic reactions. It is unclear at present whether exposures to ASDG occurs physiologically in human situation. However, there are few hypothetical situations where exposures to ASDG are possible in humans: (i) some of the alcoholic beverages contain gluten naturally (lagers, stouts, ales, and wheat beer). Therefore, in principle, such beverages have the potential to elicit sensitization via the gut as well as systemic reactions if ASDG were to enter the immune system via the gut; (ii) in the present model, skin sensitization was used as an experimental system to bypass the gastrointestinal immune tolerance that is innate in mice; in this model, ASDG was used without causing any damage to the skin; there are a number of cosmetic skin products (shampoo, moisturizer, soap, hand sanitizers, conditioners, etc.) that contain industrially processed gluten, and such gluten has been known to elicit wheat allergy (3, 60, 61). In addition, there are also after-shave lotions, and conditioners, containing both alcohol and gluten in the same product, thus making skin exposure to alcohol soluble gluten a possibility; and (iii) with the widespread use of alcohol sanitizers due to COVID-19 pandemic, whether concurrent use of excessive alcohol sanitizers along with gluten-containing skin lotions/cosmetics, makes it possible for alcohol-soluble gluten to enter the human skin remains to be tested. These are clearly speculated hypothetical situations that need further study.

Animal models used to study gluten allergenicity have been reviewed recently (2, 3). Typically, in animal models such as mice, sensitization is done by repeated IP injections of the gluten along with alum adjuvant; and the challenges are done by IP injections with the gluten protein alone. Our goal in this study was to test the utility of a non-invasive skin exposure method to induce sensitization to gluten. Therefore, we used skin application of gluten protein without tape-stripping of the stratum corneum. In order to evaluate whether skin-sensitization results in clinical sensitization for systemic anaphylaxis, we challenged the skin-sensitized mice with gluten using the systemic route of challenge (that is IP injection). Our results demonstrate that this approach can provide us the data to confirm clinical sensitization of skin-sensitized mice for systemic anaphylaxis.

One previous study in Balb/c mice evaluated the consequence of 4 weekly exposures to gluten via deliberately damaged skin by tape-stripping the stratum corneum (33). They found that gluten alone was unable to elicit IgE after 4 exposures, and unable to clinically sensitize mice and cause anaphylaxis to be triggered. However, application of gluten in the presence of a detergent (sodium dodecyl sulfate) did elicit an IgE response by 4 exposures and produce clinical sensitization. There are two major differences between our study and this previous report that may explain the discrepancy in IgE responses: (i) we tested chronic exposures (9 exposures vs. 4 exposures); and we detected IgE even after 4 exposures itself; (ii) we did not deliberately create the skin wound before exposing to gluten vs. previous researchers removed stratum corneum using tape-stripping method and then applied gluten over the damaged skin; whether deliberate damage to the skin via tape-stripping prevents IgE response to four applications of gluten in the previous study remains to be tested.

In preliminary studies we determined the IgE responses on a bi-weekly basis because of the requirement to have at least 10 days between two bleedings per animal guidelines. We determined the specific IgE antibody responses upon transdermal exposures and found a progressive increase in the specific IgE antibody response upon chronic exposure to ASDG (Supplementary Figures 6). A primary goal of this study was to determine the consequences of chronic exposure to gluten on IgE responses. Therefore, we tested the 9-time skin exposures as a model of chronic exposure to gluten. Furthermore, we also sought to saturate the system with as high IgE antibodies as possible, so that robust anaphylaxis readout would be established. As evident from HSR data, dramatic anaphylactic reactions were achieved. It is noteworthy that most previous studies of gluten allergenicity had typically used 4-time IP exposures with alum to elicit sensitization (2). Here our goal was to establish robust IgE and anaphylactic responses to chronic skin exposure to gluten without adjuvant and without injections. It is noteworthy that because significant sensitizations are elicited as early as after 4-time skin exposures, other investigators interested in short-term exposure studies may also be able to use this model for their specific needs.

Fujii et al. (2009) reported the effects of intradermal administration of 20 μL/site of 0.01%, 0.1%, and 1% ethanol solution on non-specific scratching responses in a dermatitis prone mice strain fed with a special diet that causes dermatitis in that model (62). They report that all doses of ethanol administered intradermally failed to induce a scratching response in mice fed with both normal diet- and the HR-AD-fed mice that develop dermatitis. Thus, they show that alcohol application in fact protects mice from scratching response in HR-AD-fed that normally develop scratching. Notably, in that model dermatitis develops upon feeding this diet, and not by allergen application. There is no involvement of IgE antibody responses to allergens in this model. In contrast, in our model, Balb/c mice are used and IgE antibody responses to ASDG allergen application over the skin is studied. Both are different types of studies and therefore side-by-side comparison is not feasible.

In our study, ASDG was dissolved in 70% ethanol and applied over a small area (2 cm^2^) of rump skin to elicit specific-IgE antibody responses because gliadin protein requires alcohol for solubilization. Because of this, we have used the term “alcohol-soluble durum gluten” (ASDG) in this paper. It is important to note that we are not studying the intrinsic allergenicity of gliadin *per se* because it is an insoluble protein in aqueous solutions. Rather, here we are reporting the intrinsic allergenicity of “alcohol-solubilized gliadin protein”. Therefore, whether 70% ethanol, that is required for solubilizing the gliadin protein, might act as an adjuvant for specific IgE antibody responses to gliadin remains unknown. Technically, it is not possible to apply ASDG over the skin in aqueous solutions such as PBS in a consistently dose-controlled manner as it becomes insoluble and forms a viscous suspension. Also, we are not aware of any previous published studies suggesting that 70% ethyl alcohol has any adjuvant activity on allergic responses in mouse models. Since typical allergens such as ovalbumin are not soluble in 70% ethanol it is not possible to test whether ethanol might act as adjuvant for ovalbumin or any other aqueous soluble protein allergens in skin sensitization model since applied insoluble material does not stay in place and measurement of responses in a dose-controlled manner are technically not feasible. There is one interesting study that tests the effect of dodecyl alcohol ethoxylate (a nonionic surfactant derived from alcohol) on specific IgE response to ovalbumin in Balb/cA mice (63). They reported that there was no significant impact of dodecyl alcohol on IgE responses in general except that at one dose there was inhibitory effect (63).

There is some evidence in humans that sensitization to gluten on to undamaged skin can occur via usage of cosmetic such as facial soap (60, 61). In these reports, authors demonstrate that facial soaps containing Glupearl 19S (an acid hydrolyzed wheat protein) caused sensitization of several Japanese subjects that subsequentially developed anaphylaxis or urticaria upon consumption of wheat-containing food products. Thus, our results in mouse model further support the hypothesis that exposure to gluten via undamaged skin may have the potential to sensitize humans.

In this study we measured the systemic *in vivo* levels of cytokines in the spleen extracts (which is a crude way of measurement) because our goal was to identify the cytokine biomarkers associated with systemic anaphylaxis that are elevated by 1 h-after systemic ASDG challenge. The rationale for this objective was that such cytokines might as biomarkers of life-threatening systemic anaphylaxis. We have identified that a few cytokines are significantly elevated during the systemic anaphylaxis reaction in this model. Our goal was not to study recall cytokine responses in this model. Recall cytokine responses to gliadin in Balb/c mice has been previously reported (64).

In this study, we report cytokine productions *in vivo* in the spleen of mice undergoing systemic anaphylaxis. However, the cellular source of various cytokines remains to be determined. Due to limited resources, and focus on the current grant funding, we have not been able to conduct flow cytometry studies at this point. However, it is important to conduct such studies. We plan to do detailed flow cytometry analysis to identify the cellular contributors of cytokine production observed in the spleen in future studies.

Multiple parameters were evaluated during the current study, providing detailed characterization of the mouse model for gluten sensitization and anaphylaxis, aspects that previous animal model studies have not reported on. These include: (i) systemic anaphylaxis to wheat gluten is associated with mucosal mast cell degranulation responses (MMCRs), quantifiable by measuring MMCP-1 levels in the blood which is a specific biological marker of IgE antibody-mediated systemic anaphylaxis in mice (40); (ii) using single mouse data we conducted analyses and demonstrated correlations among the 3 quantifiable and objective readouts of anaphylaxis (IgE, HSR and MMCR); (iii) using spleen cells, we characterized the Th1, and Th2 cytokine profile of mice undergoing life-threatening systemic anaphylaxis; and (iv) we also analyzed a large panel of other immune markers (chemokines, and adhesion molecules) and identified those that are acutely and substantially elevated (2.5 to 43-fold) during the clinical symptoms of systemic anaphylaxis. It is noteworthy that most of these immune markers have been previously linked to allergic and Th2 immune responses (65–69). Thus, our study has significantly advanced the mouse model development and characterization efforts to improve the current models of gluten allergy, particularly using an adjuvant-free approach to elicit gluten hypersensitivity. Furthermore, our approach of conducting exposures to gluten on intact murine skin, without deliberately causing skin-damage by tape-stripping, has also advanced the humane use of animals for model development.

Hypothermic shock responses (HSRs) were measured by rectal thermometry before challenge and at every 5 min up to 30 min after challenge. At one hour after challenge, mice were bled, and plasma was used to measure MMCP-1 levels. Measurement of MMCP-1 in the blood at 1 h after challenge reflects anaphylaxis caused by IgE antibody mediated mucosal mast cell degranulation response in mice (40). The HSR represents the consequence of anaphylaxis on neurological and cardiovascular functions involved in thermoregulation. Our goal was to determine whether mucosal mast cell response was associated with HSR noted upon systemic challenge. Our data show that there is a significant co-relationship between the HSR and MMCP-1 responses–the two objective and quantitative markers of anaphylaxis. This explains that the underlying mechanism of gluten-induced anaphylaxis as quantified by HSR involves participation of IgE antibody mediated activation and degranulation of mucosal mast cells in this model.

The gastrointestinal tract (gut) contains mucosal mast cells that are filled with MMCP-1 in their granules, which is a characteristic granule protein of only mucosal mast cells, and it is absent in connective tissue mast cells. The gut mucosal mast cells have receptors for IgE antibodies. Upon sensitization to gluten, IgE antibodies bind to mucosal mast cells via the high affinity IgE antibody receptors (Fc*ε*RI). At this point, blood has very little background levels of MMCP-1 as reflected in pre-challenge samples because the mucosal mast cells are not activated. Upon challenge, gluten binds to gut mast cells associated IgE antibody leading to activation and degranulation of mast cells, thus releasing MMCP-1 into the systemic circulation. Elevation of MMCP-1 at one-hour timepoint after challenge indicates IgE antibody mediated anaphylaxis in the gut as reported previously (40). Therefore, demonstrating significant elevation of MMCP-1 in the blood at 1-h after challenge represents IgE antibody mediated mucosal mast cell degranulation response associated with systemic anaphylaxis in this model.

The most common quantitative parameters used to study allergic response in mouse models food allergy are food specific IgE antibody levels in the blood, elevation of MMCP-1 upon allergen challenge in the blood, and significant drop in the rectal temperature upon allergen challenge (2, 3). One of the aims of the current study was to test the relationships among quantitative parameters associated with systemic anaphylaxis in this model and validate them for testing changes to gluten allergenicity by processing methods and genetic modifications in the future. The rationale underlying this idea is that it will inform the mechanisms leading to life-threatening anaphylaxis. As we found, there is a significant correlation among the three quantitative parameters, which suggests that the underlying mechanism of systemic anaphylaxis as well as hypothermic shock response observed in this model involves specific IgE antibody mediated activation of mucosal mast cells resulting in degranulation of mediators of anaphylaxis. Therefore, these data validate the future use of all the three readouts for testing impact of modification of gluten allergenicity by processing methods and genetic approaches in future research studies.

Gluten hypersensitivity has been studied in a number of animal models previously, including dog, rat, guinea pig and mice (27–31, 70). In dog models, a genetic breed variant was identified to develop wheat allergy along with allergy to other foods when wheat flour was administered by injections along with adjuvants followed by repeated oral challenges to elicit diarrhea (27). They demonstrated that not only gluten, but also non-gluten proteins elicited IgE responses leading to skin sensitization as measured by skin prick testing with respective proteins. Based on their results, we made some improvements in indicators of wheat allergenicity, such as life-threatening wheat anaphylaxis, correlations among allergy readouts, and characterized cytokine and other immune response markers (71).

In rat models, consequences of both the oral route of exposure as well as the skin route of exposure to gluten have been reported (30, 70). Oral exposure to gluten was shown to enhance allergic sensitization (IgE) capacity only with enzyme-hydrolyzed gluten. They also found that acid hydrolysis of gluten results in the generation of novel IgG binding epitopes (70). This study had focused on specific IgE, IgG, and rat basophilic leukemia cell degranulation *in vitro* as the readouts of disease. In the skin exposure rat model, researchers used sandpaper to cause skin injury similar to the use of tape-stripping to remove stratum corneum in the previous mouse model discussed above (33). In mouse model of systemic anaphylaxis induced by intraperitoneal injections, specific IgG1 antibodies participate in reactions (72). Therefore, we measured ASDG-specific IgG1 antibodies in this model and our data demonstrate robust IgG1 responses in this model.

Several studies have reported on mouse models used to study gluten hypersensitivity (59, 64, 73–76); all except one used alum adjuvant to elicit IgE responses to gluten upon intraperitoneal injections, the exception used detergent as adjuvant to elicit sensitization upon application over damaged skin by tape-stripping of stratum corneum. Key endpoints used in these studies were as follows: specific IgE and total IgE, HSR, and symptom scoring. None of the studies determined MMCP-1 responses. It should be noted that neither correlation studies among allergenicity markers nor systemic immune marker analysis associated with anaphylaxis have been reported before in gluten hypersensitivity mouse models.

Notably, of the previous animal models of gluten hypersensitivity investigated, none have reported systemic immune markers associated with clinical symptoms of systemic anaphylaxis. We found that selected Th2 markers are positively associated with anaphylaxis and selected Th1 markers are negatively associated with anaphylaxis. Furthermore, the large panel of other novel immune markers found to be associated with anaphylaxis were also identified in this study. Therefore, these data suggest the complexity of the acute adverse immune response elicited by gluten in the ASDG-sensitized murine host. Thus, these markers' relevance can be tested in human gluten anaphylaxis and their suitability for monitoring the severity of the disease can be determined in future. Furthermore, they may also provide leads to developing novel therapeutics targeting the common pathways leading to their activation during the life-threatening anaphylactic response to gluten.

Previous elegant studies have identified specific gluten allergens in humans as follows: α-, γ-, ω-2, and ω-5 gliadins and low/high molecular weight glutenin subunits (4, 59, 77). Furthermore, epitope mapping demonstrated that IgE epitopes on these allergens are very similar between wheat allergic humans and Balb/c mice that are sensitized to wheat (59). Previously, Jorgensen et al., demonstrated that non-gluten salt-soluble allergens in sensitized Balb/c mice are also reported as major allergens in humans allergic to wheat (78). Thus, Balb/c mouse model represents a very valuable experimental system to study human wheat allergenicity.

Thus, the mouse model we report here is not only significantly different from previous reported models, but it also makes further improvements to the existing models so that the following novel and critical questions can be addressed effectively in the future which was not possible previously: (i) study the intrinsic allergenicity of glutens from wheat lines that are genetically different in ploidy, as well as compare the intrinsic allergenicity of various wheat lines within diploid, tetraploid and hexaploid wheats; this approach has the potential to identify hypo/non/hyper-allergenic wheats for future consumer and medical uses; (ii) determine the intrinsic allergenicity potential of genetically engineered (GE) wheats to establish substantial equivalence with the native wheat line as part of evaluating the food safety of GE foods as recommended by the Food and Agriculture Organization/World Health Organization in their flow chart model for this purpose (79); (iii) determine the impact of processing on gluten allergenicity so that effects of food and industrial processing on gluten allergenicity can be quantitatively assessed to protect consumers of products from both the wheat food industry and the cosmetic skin health care industry (3); (iv) the pre-clinical adjuvant-free mouse model reported here will also be useful in developing novel dietary intervention and therapeutic and vaccine approaches for gluten hypersensitivity.

This study collectively demonstrates that ASDG is intrinsically allergenic; and chronic exposure to ASDG via undamaged skin may clinically sensitize mice for life-threatening anaphylaxis via activating the systemic Th2 immune response. In conclusion, we report the development and characterization of a novel mouse model of gluten hypersensitivity that has significantly advanced the animal model research on gluten allergenicity.

## Data Availability

The original contributions presented in the study are included in the article/**Supplementary Material**, further inquiries can be directed to the corresponding author.
