# Supplementary material for: Chronic application of alcohol-soluble gluten extract over undamaged skin causes clinical sensitization for life-threatening anaphylaxis via activation of systemic Th2 immune responses in mice
Source: Front Allergy. 2023 Sep 29;4:1214051. doi: 10.3389/falgy.2023.1214051 (PMC10570422; doi:10.3389/falgy.2023.1214051)
Supplement: Supplementary file 1 [file Presentation1.pptx]

## Slide 1
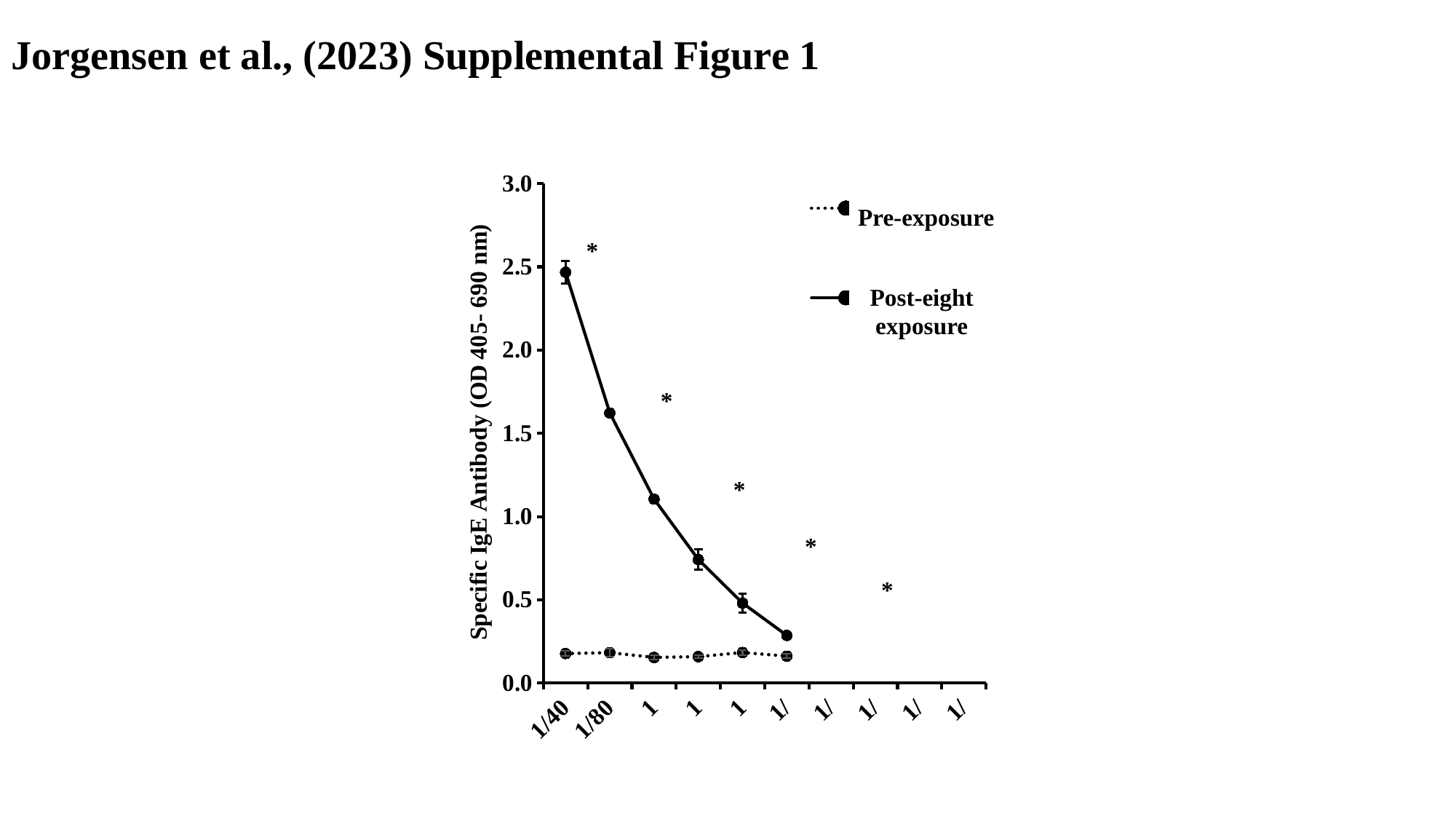

Jorgensen et al., (2023) Supplemental Figure 1
[unsupported chart]
Pre-exposure
*
Post-eight exposure
*
*
*
*

## Slide 2
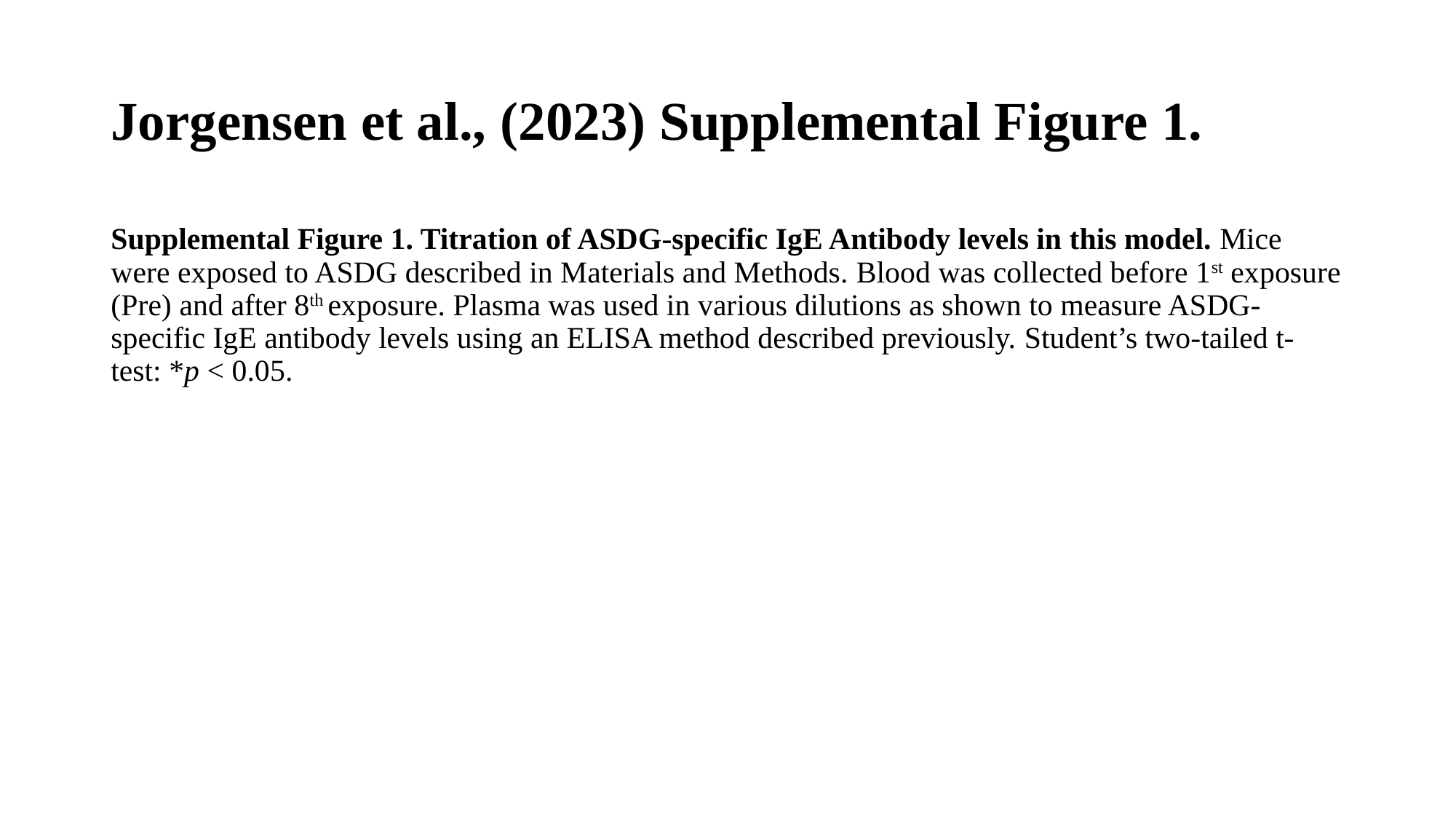

# Jorgensen et al., (2023) Supplemental Figure 1.
Supplemental Figure 1. Titration of ASDG-specific IgE Antibody levels in this model. Mice were exposed to ASDG described in Materials and Methods. Blood was collected before 1st exposure (Pre) and after 8th exposure. Plasma was used in various dilutions as shown to measure ASDG-specific IgE antibody levels using an ELISA method described previously. Student’s two-tailed t-test: *p < 0.05.

## Slide 3
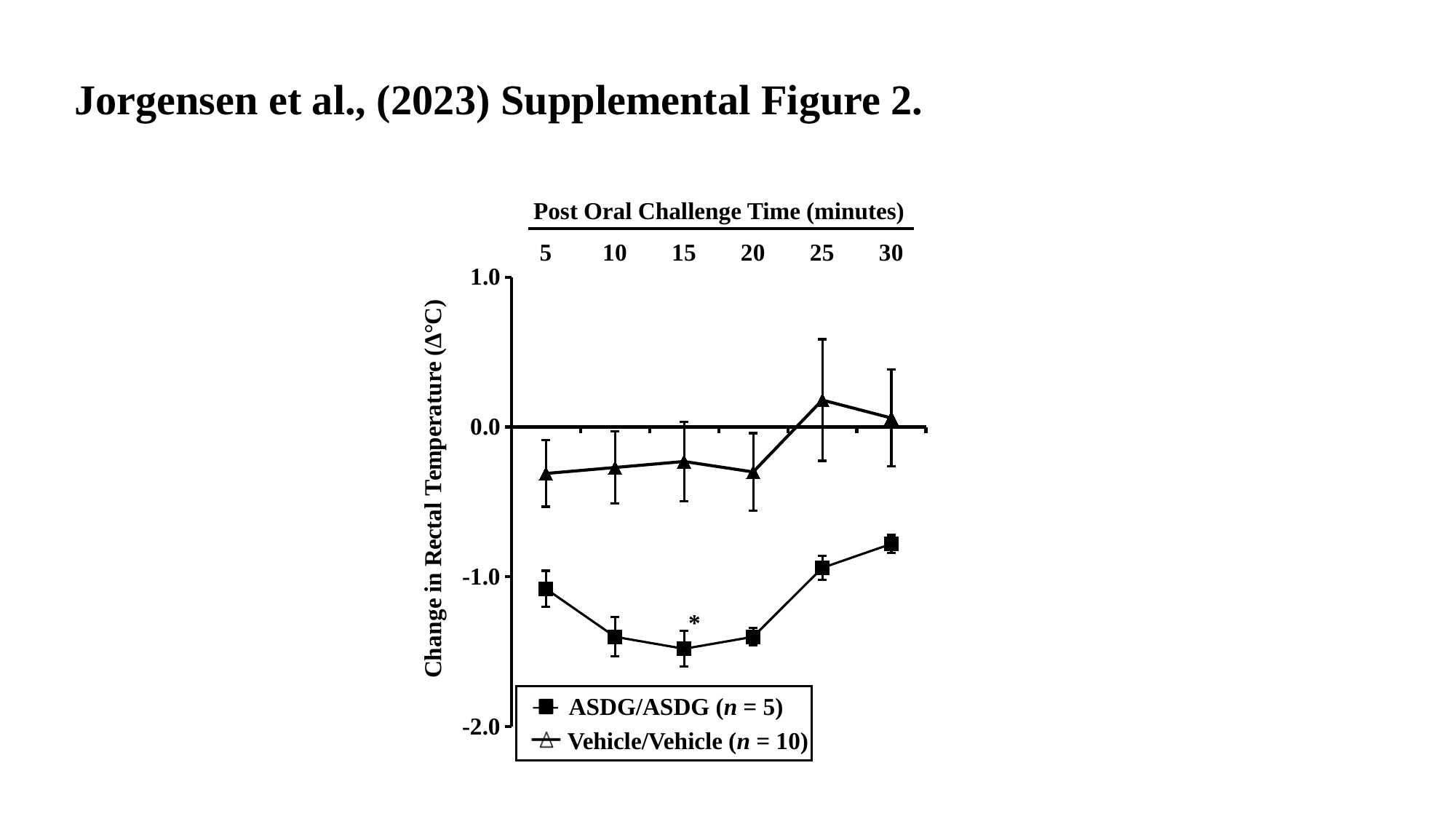

Jorgensen et al., (2023) Supplemental Figure 2.
Post Oral Challenge Time (minutes)
### Chart
| Category | | |
|---|---|---|
| 5 | -0.30999999999999517 | -1.0799999999999983 |
| 10 | -0.269999999999996 | -1.3999999999999986 |
| 15 | -0.22999999999999687 | -1.4799999999999969 |
| 20 | -0.29999999999999005 | -1.4000000000000057 |
| 25 | 0.17999999999999972 | -0.9400000000000048 |
| 30 | 0.060000000000002274 | -0.7800000000000082 |*
ASDG/ASDG (n = 5)
Vehicle/Vehicle (n = 10)

## Slide 4
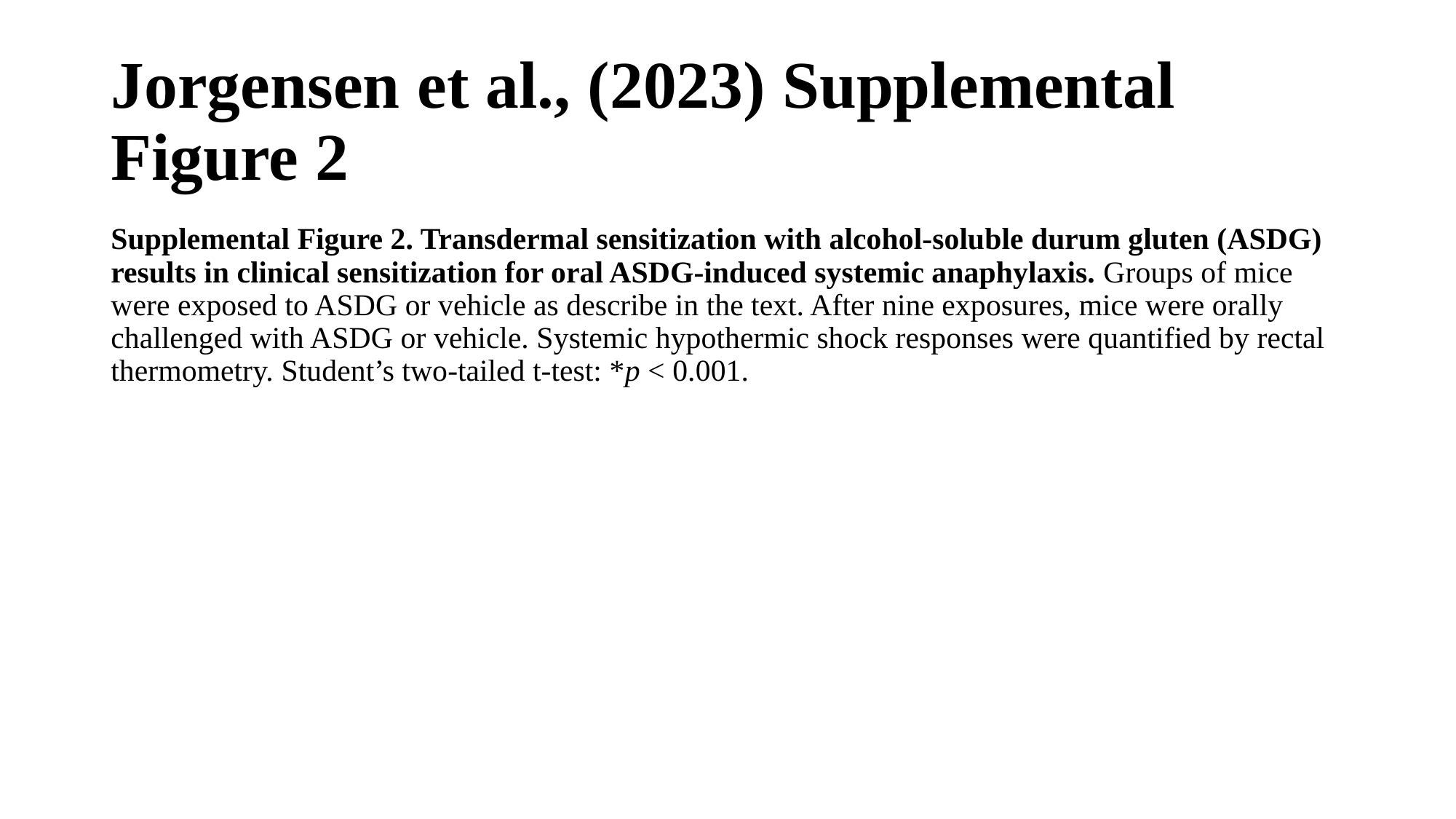

# Jorgensen et al., (2023) Supplemental Figure 2
Supplemental Figure 2. Transdermal sensitization with alcohol-soluble durum gluten (ASDG) results in clinical sensitization for oral ASDG-induced systemic anaphylaxis. Groups of mice were exposed to ASDG or vehicle as describe in the text. After nine exposures, mice were orally challenged with ASDG or vehicle. Systemic hypothermic shock responses were quantified by rectal thermometry. Student’s two-tailed t-test: *p < 0.001.

## Slide 5
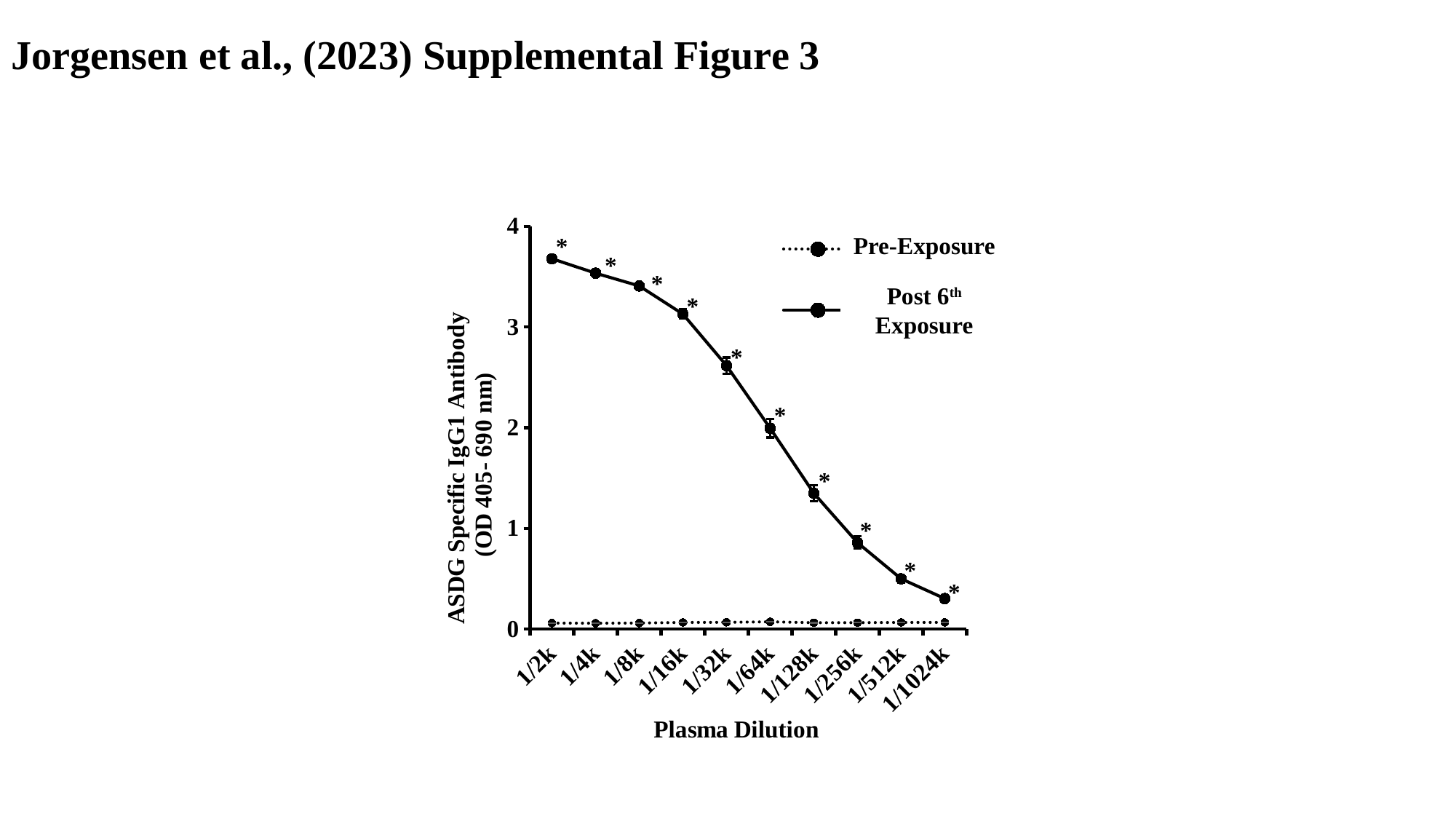

Jorgensen et al., (2023) Supplemental Figure 3
Pre-Exposure
Post 6th Exposure
### Chart
| Category | ASDG Pre (n=20) | ASDG 6R (n=20) |
|---|---|---|
| 1/2k | 0.06075 | 3.6777499999999996 |
| 1/4k | 0.0595 | 3.5344999999999995 |
| 1/8k | 0.06125 | 3.40775 |
| 1/16k | 0.067 | 3.1285 |
| 1/32k | 0.0685 | 2.6165 |
| 1/64k | 0.07350000000000001 | 1.9949999999999999 |
| 1/128k | 0.06475 | 1.35 |
| 1/256k | 0.06525 | 0.8594999999999999 |
| 1/512k | 0.06725 | 0.5005000000000001 |
| 1/1024k | 0.06775 | 0.3045 |
*
*
*
*
*
*
*
*
*
*

## Slide 6
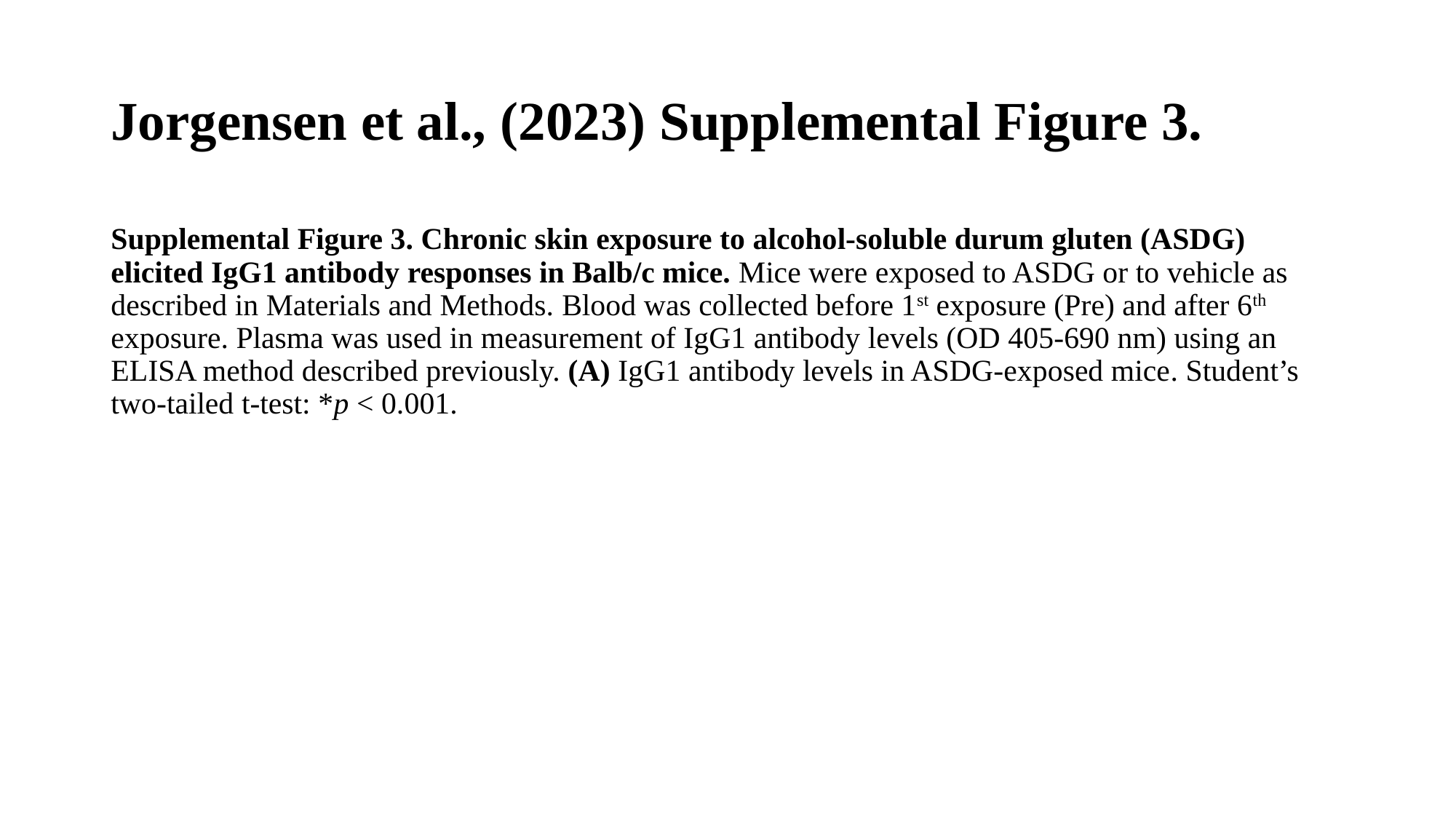

# Jorgensen et al., (2023) Supplemental Figure 3.
Supplemental Figure 3. Chronic skin exposure to alcohol-soluble durum gluten (ASDG) elicited IgG1 antibody responses in Balb/c mice. Mice were exposed to ASDG or to vehicle as described in Materials and Methods. Blood was collected before 1st exposure (Pre) and after 6th exposure. Plasma was used in measurement of IgG1 antibody levels (OD 405-690 nm) using an ELISA method described previously. (A) IgG1 antibody levels in ASDG-exposed mice. Student’s two-tailed t-test: *p < 0.001.

## Slide 7
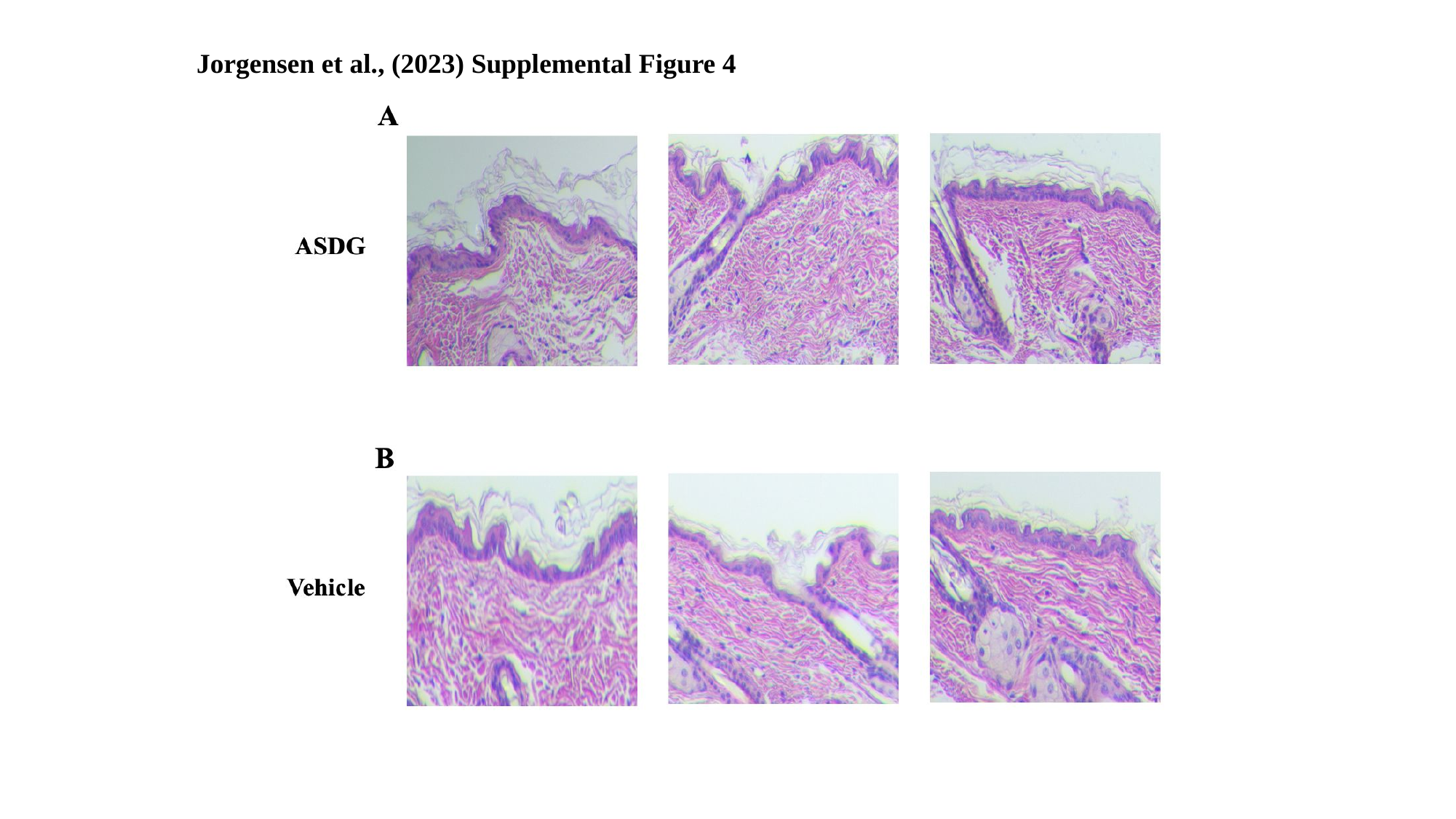

Jorgensen et al., (2023) Supplemental Figure 4

## Slide 8
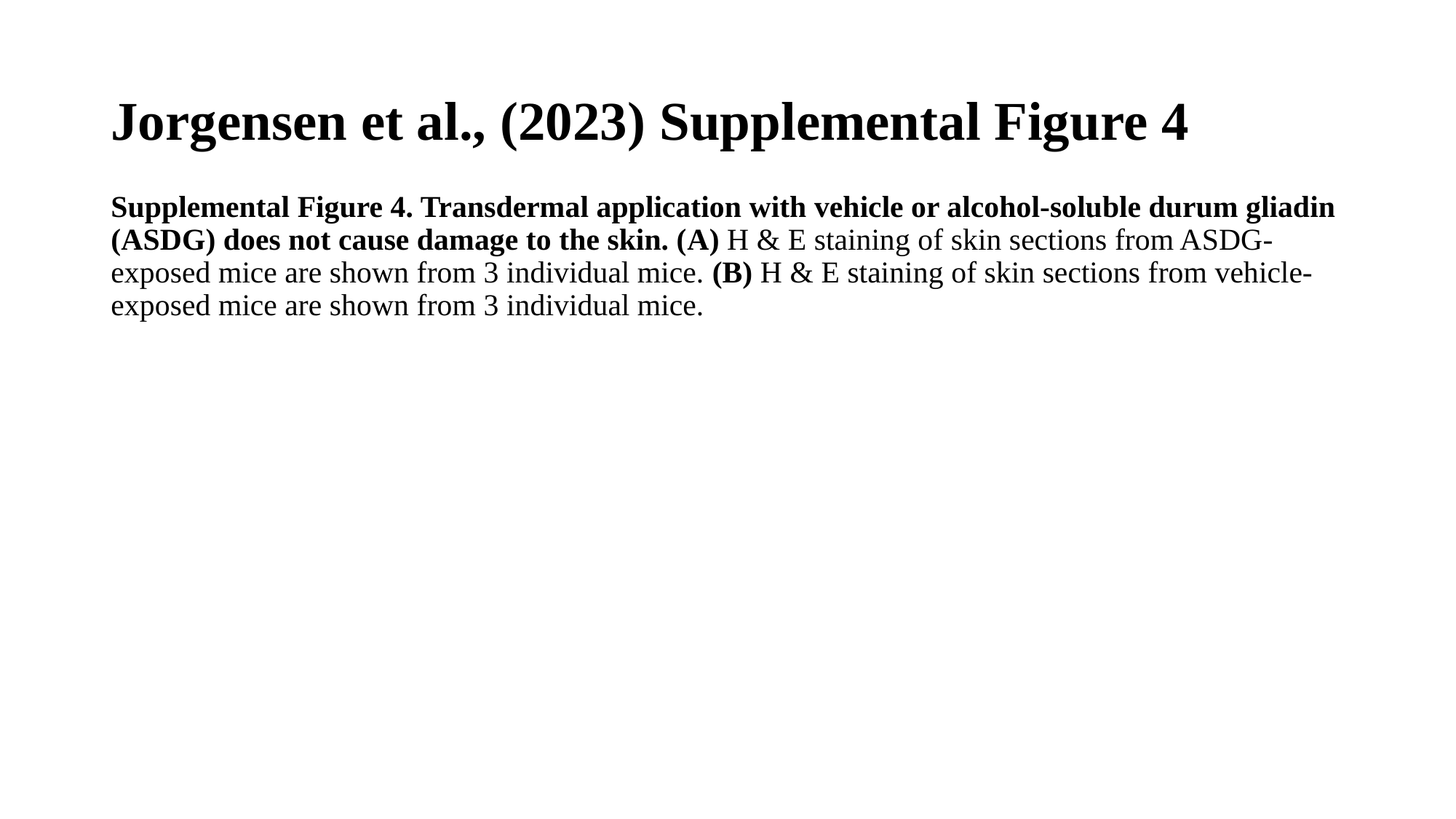

# Jorgensen et al., (2023) Supplemental Figure 4
Supplemental Figure 4. Transdermal application with vehicle or alcohol-soluble durum gliadin (ASDG) does not cause damage to the skin. (A) H & E staining of skin sections from ASDG-exposed mice are shown from 3 individual mice. (B) H & E staining of skin sections from vehicle-exposed mice are shown from 3 individual mice.

## Slide 9
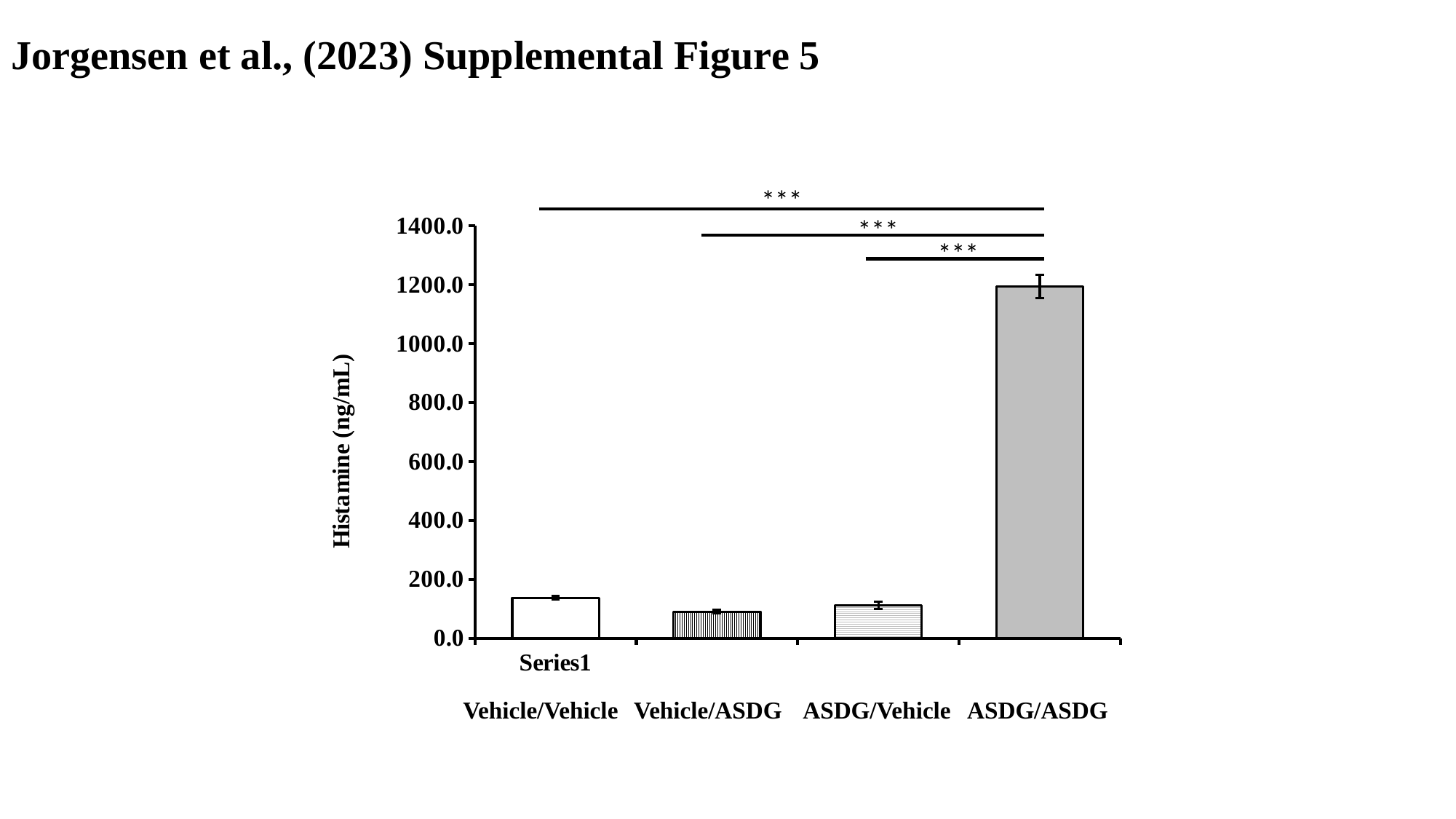

Jorgensen et al., (2023) Supplemental Figure 5
***
### Chart
| Category | |
|---|---|***
***
Vehicle/Vehicle
Vehicle/ASDG
ASDG/Vehicle
ASDG/ASDG

## Slide 10
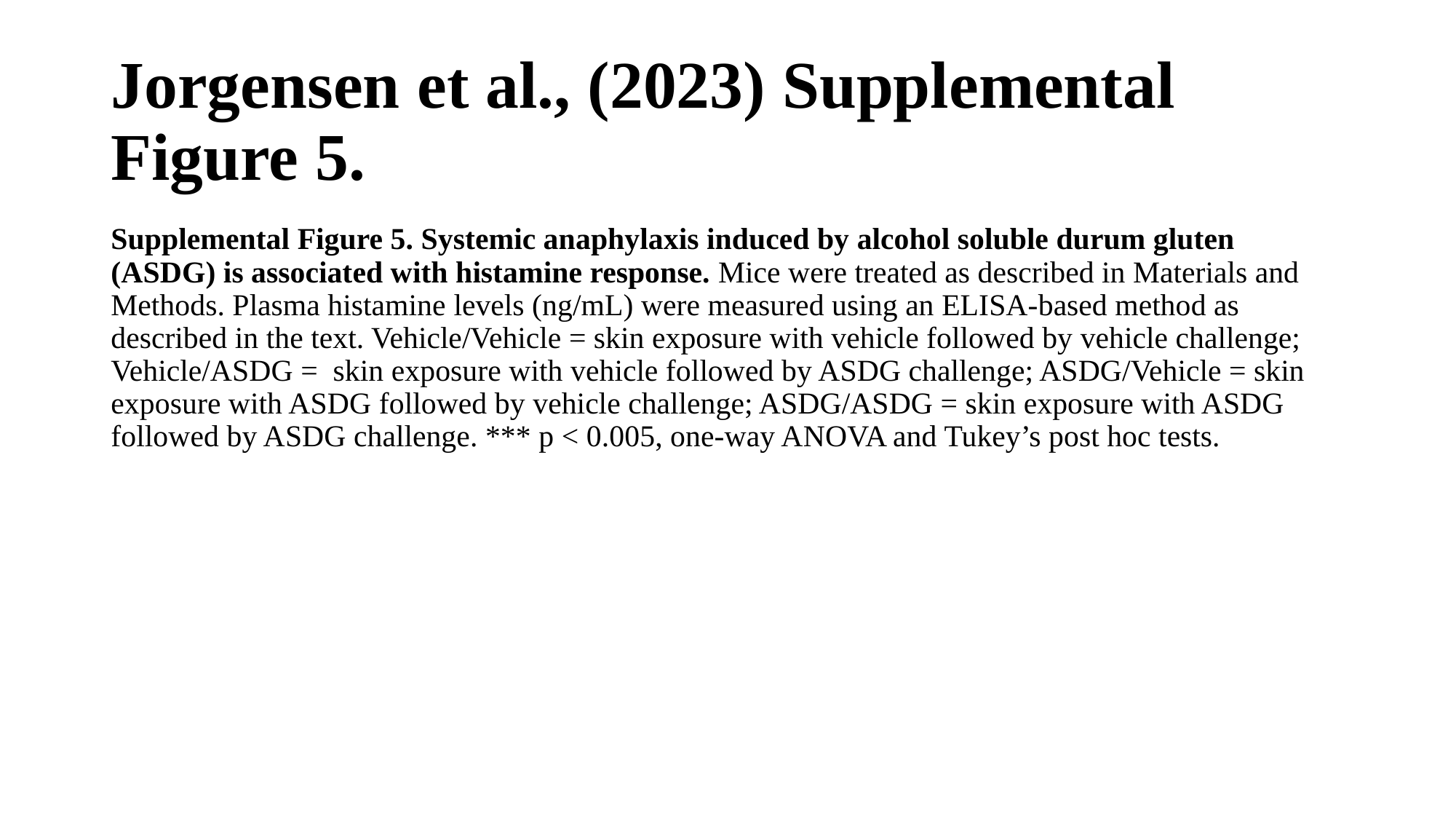

# Jorgensen et al., (2023) Supplemental Figure 5.
Supplemental Figure 5. Systemic anaphylaxis induced by alcohol soluble durum gluten (ASDG) is associated with histamine response. Mice were treated as described in Materials and Methods. Plasma histamine levels (ng/mL) were measured using an ELISA-based method as described in the text. Vehicle/Vehicle = skin exposure with vehicle followed by vehicle challenge; Vehicle/ASDG = skin exposure with vehicle followed by ASDG challenge; ASDG/Vehicle = skin exposure with ASDG followed by vehicle challenge; ASDG/ASDG = skin exposure with ASDG followed by ASDG challenge. *** p < 0.005, one-way ANOVA and Tukey’s post hoc tests.

## Slide 11
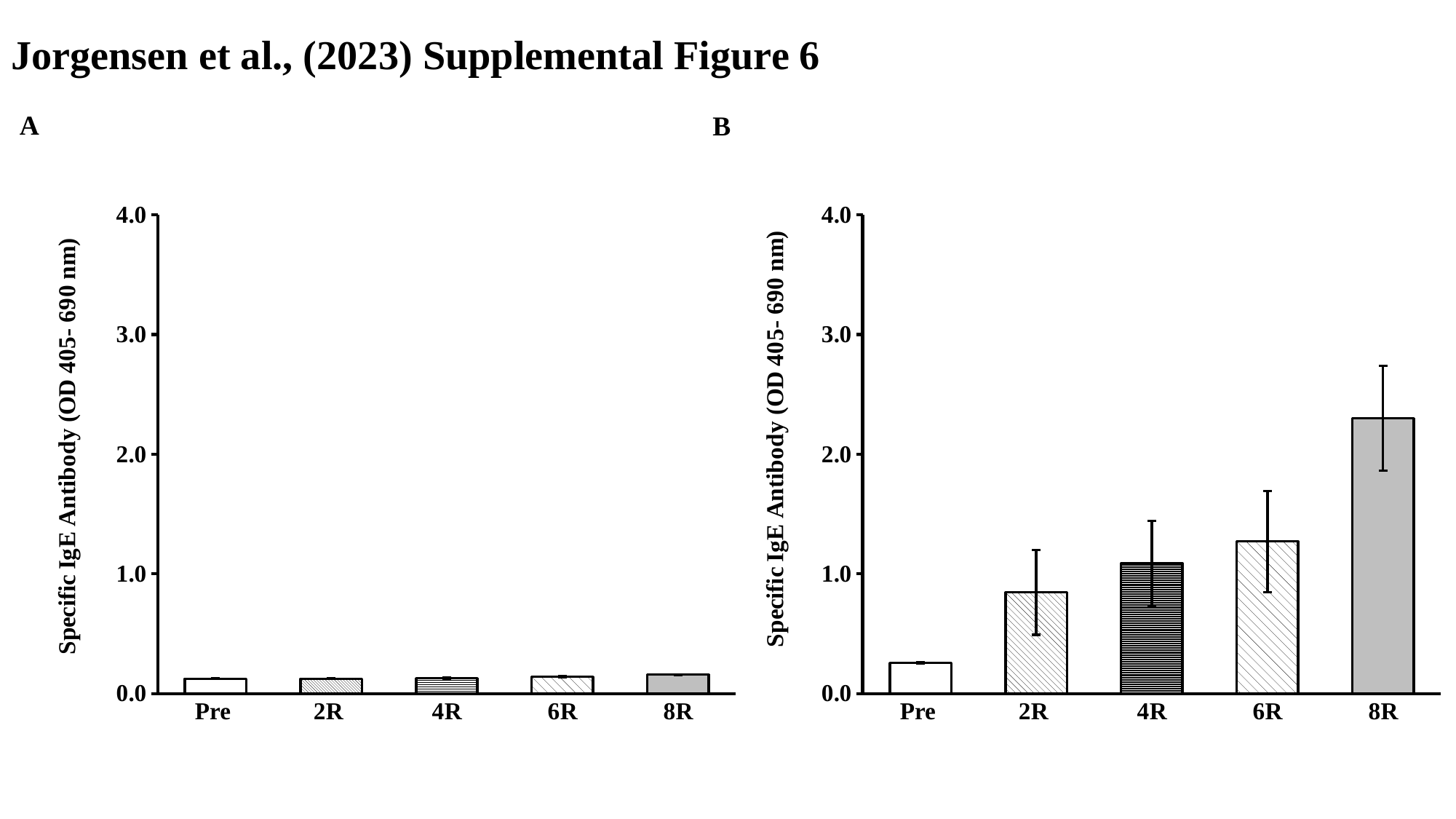

Jorgensen et al., (2023) Supplemental Figure 6
A
B
### Chart
| Category | Pre |
|---|---|
| Pre | 0.125925 |
| 2R | 0.12467500000000001 |
| 4R | 0.12875 |
| 6R | 0.14042500000000002 |
| 8R | 0.158 |
### Chart
| Category | Pre |
|---|---|
| Pre | 0.25637499999999996 |
| 2R | 0.8444249999999999 |
| 4R | 1.08825 |
| 6R | 1.270975 |
| 8R | 2.3 |

## Slide 12
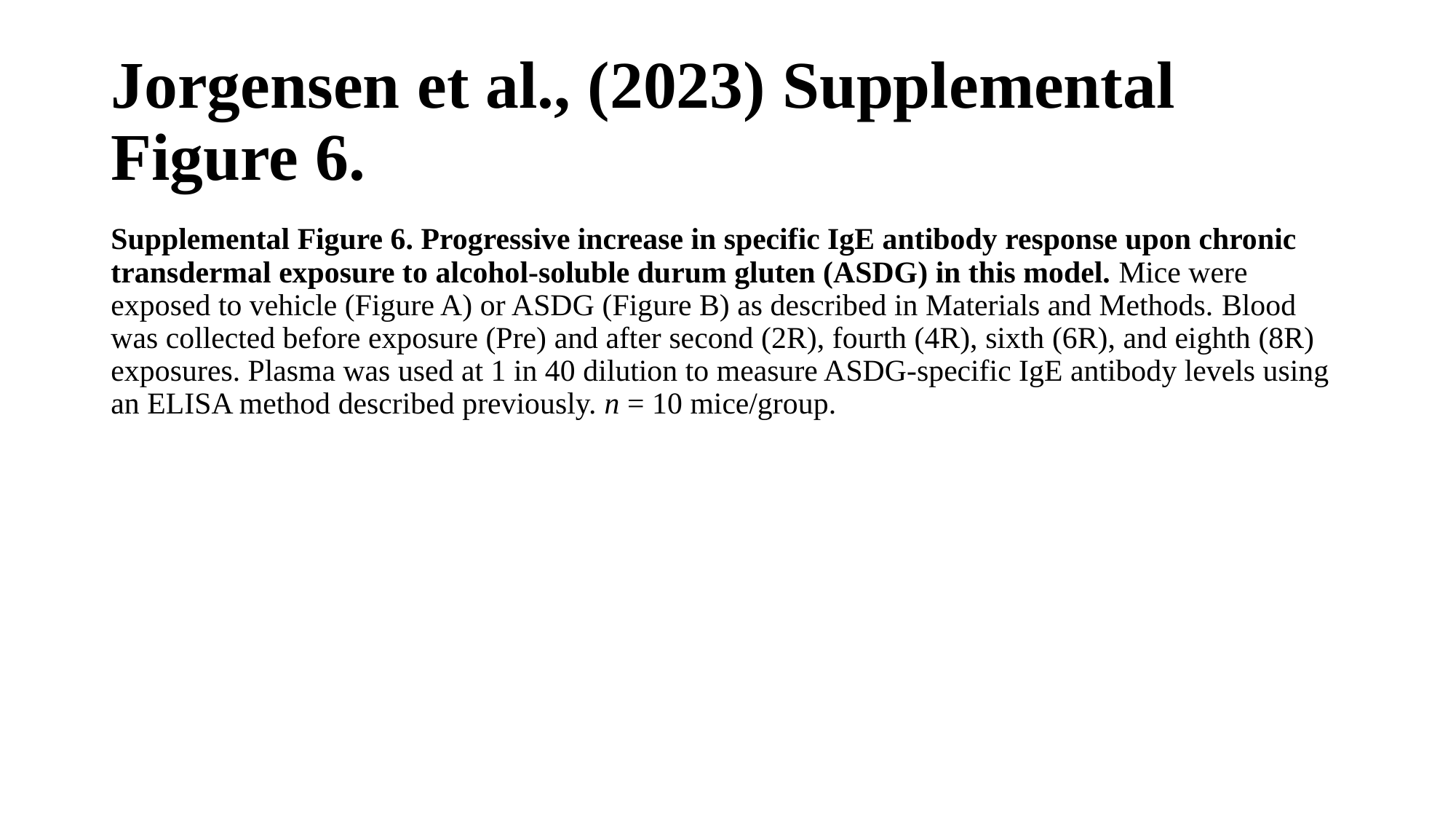

# Jorgensen et al., (2023) Supplemental Figure 6.
Supplemental Figure 6. Progressive increase in specific IgE antibody response upon chronic transdermal exposure to alcohol-soluble durum gluten (ASDG) in this model. Mice were exposed to vehicle (Figure A) or ASDG (Figure B) as described in Materials and Methods. Blood was collected before exposure (Pre) and after second (2R), fourth (4R), sixth (6R), and eighth (8R) exposures. Plasma was used at 1 in 40 dilution to measure ASDG-specific IgE antibody levels using an ELISA method described previously. n = 10 mice/group.
